# Coronavirus Gene 7 Counteracts Host Defenses and Modulates Virus Virulence

**DOI:** 10.1371/journal.ppat.1002090

**Published:** 2011-06-09

**Authors:** Jazmina L. G. Cruz, Isabel Sola, Martina Becares, Berta Alberca, Joan Plana, Luis Enjuanes, Sonia Zuñiga

**Affiliations:** 1 Centro Nacional de Biotecnología, CNB, CSIC, Department of Molecular and Cell Biology, Darwin 3, Campus Universidad Autónoma de Madrid, Cantoblanco, Madrid, Spain; 2 Pfizer Animal Health, Girona, Spain; University of North Carolina at Chapel Hill, United States of America

## Abstract

Transmissible gastroenteritis virus (TGEV) genome contains three accessory genes: 3a, 3b and 7. Gene 7 is only present in members of coronavirus genus a1, and encodes a hydrophobic protein of 78 aa. To study gene 7 function, a recombinant TGEV virus lacking gene 7 was engineered (rTGEV-Δ7). Both the mutant and the parental (rTGEV-*wt*) viruses showed the same growth and viral RNA accumulation kinetics in tissue cultures. Nevertheless, cells infected with rTGEV-Δ7 virus showed an increased cytopathic effect caused by an enhanced apoptosis mediated by caspase activation. Macromolecular synthesis analysis showed that rTGEV-Δ7 virus infection led to host translational shut-off and increased cellular RNA degradation compared with rTGEV-*wt* infection. An increase of eukaryotic translation initiation factor 2 (eIF2α) phosphorylation and an enhanced nuclease, most likely RNase L, activity were observed in rTGEV-Δ7 virus infected cells. These results suggested that the removal of gene 7 promoted an intensified dsRNA-activated host antiviral response. In protein 7 a conserved sequence motif that potentially mediates binding to protein phosphatase 1 catalytic subunit (PP1c), a key regulator of the cell antiviral defenses, was identified. We postulated that TGEV protein 7 may counteract host antiviral response by its association with PP1c. In fact, pull-down assays demonstrated the interaction between TGEV protein 7, but not a protein 7 mutant lacking PP1c binding motif, with PP1. Moreover, the interaction between protein 7 and PP1 was required, during the infection, for eIF2α dephosphorylation and inhibition of cell RNA degradation. Inoculation of newborn piglets with rTGEV-Δ7 and rTGEV-*wt* viruses showed that rTGEV-Δ7 virus presented accelerated growth kinetics and pathology compared with the parental virus. Overall, the results indicated that gene 7 counteracted host cell defenses, and modified TGEV persistence increasing TGEV survival. Therefore, the acquisition of gene 7 by the TGEV genome most likely has provided a selective advantage to the virus.

## Author Summary

Innate immune response is the first line of antiviral defense. Viruses have developed diverse strategies to evade this deleterious response, ensuring their survival. Several CoV accessory genes play a central role in these pathways. Nevertheless, the molecular mechanisms by which they exert their function are still unknown. The generation of a rTGEV without gene 7 expression allowed us to study the role of protein 7 in the modulation of the antiviral response. The absence of protein 7 during TGEV infection caused an enhanced apoptosis and a translational shutoff, due to an increased cellular RNA degradation and eIF2α phosphorylation. We identified a protein phosphatase 1 (PP1) binding motif in protein 7, and a TGEV protein 7-PP1 interaction was demonstrated. We propose a novel mechanism to counteract dsRNA-induced antiviral response by RNA viruses. *In vit*ro results were in agreement with the enhanced virulence of the gene 7 deletion mutant virus in infected piglets. Our results demonstrated that protein 7 modifies TGEV virulence, reducing virus pathology and increasing the period of virus shedding. This effect also benefits the host decreasing clinical disease and extending its survival. These observations could justify the incorporation and maintenance of gene 7 to genus a1 CoVs during their evolution.

## Introduction

The order *Nidovirales* comprises enveloped single-stranded, positive-sense RNA viruses. The *Nidovirales* includes the *Coronaviridae* that contains viruses with the largest known RNA genome, of around 30 Kb [1], [2]. Coronaviruses (CoVs) have been classified into 3 genera, α, β and γ {de Groot, 2010 #9759}. They are the causative agents of a variety of human and animal diseases. In humans, CoVs produce respiratory tract infections, causing from the common cold to severe pneumonia and acute respiratory distress syndrome (ARDS) that may result in death [3], [4], [5]. In animals, CoVs also cause life-threatening diseases, such as severe enteric and respiratory tract infections, and are economically important pathogens [6]. Nevertheless, there is limited information about the molecular mechanisms governing CoV virulence and pathogenesis.

Double-stranded RNA (dsRNA), produced by RNA viruses as a replication intermediate, is the pathogen-associated molecular pattern that mediates the activation of a well-characterized antiviral mechanism leading to viral protein synthesis shut down [7]. This pathway includes the activation of double-stranded RNA-dependent protein kinase (PKR), leading to eukaryotic translation initiation factor 2 (eIF2α) phosphorylation, and the activation of the 2′-5′-oligoadenylate synthetase (2′-5′OAS) and its effector enzyme, the ribonuclease L (RNase L), responsible for RNA degradation [8], [9], [10], [11], [12]. Due to the deleterious effects of this response on virus survival, many viruses have developed different strategies that counteract the host antiviral response triggered by the dsRNA. These mechanisms are mediated by viral proteins or RNAs [13], [14], [15], [16], [17], [18], [19], [20], [21], [22], or by the modification of cellular components [23], [24], [25], [26], [27].

CoV replication occurs in the cytoplasm, leading to dsRNA species that trigger the host antiviral response. To overcome these defenses, CoVs have developed several strategies. A general mechanism for all CoVs is the induction of structures in infected cells that may hide viral RNAs from the cellular sensors [28], [29]. Some CoVs downregulate host gene expression. In fact, it has been proposed that genus β CoV non structural protein (nsp)1 protein promotes host mRNA degradation in order to suppress host innate immune response [30], [31]. Severe acute respiratory syndrome (SARS)-CoV nsp1 has also been involved in the inhibition of the 40S ribosomal subunit translational activity [30]. Moreover, several CoVs may also prevent the translational shutoff due to the antiviral response, using viral components or modulating cellular factors. Infectious bronchitis virus (IBV) nsp2 acts as a PKR antagonist [32], and MHV N protein antagonizes 2′-5′ OAS activity [33]. IBV also induces the over-expression of growth arrest DNA-damage 34 (GADD34) protein, which participates in eIF2α dephosphorylation [32].

The 5′ two thirds of CoV genome encode the replicase proteins that are expressed from two overlapping open reading frames (ORFs) 1a and 1b [34]. The 3′ one third of the genome contains the genes encoding structural proteins and a set of accessory genes, whose sequence and number differ between the different species of CoV [1], [35]. Traditionally, CoV accessory genes have been related to virulence modulation, such as mouse hepatitis virus (MHV) gene 5a that determines the interferon (IFN) resistance of the different MHV strains [36]. SARS-CoV contains the largest number of accessory genes and it has been proposed that these genes could be responsible for its high virulence [37], [38]. The role of some structural genes, such as SARS-CoV genes E and 6, on CoV pathogenesis has been demonstrated [39], [40], [41], [42]. Nevertheless, the role of other SARS-CoV accessory genes in virus replication and pathogenesis is still under study, as SARS-CoV mutants lacking different combinations of these genes revealed that they had limited impact on virus replication and pathogenesis [37], [38].

**Figure 1:**
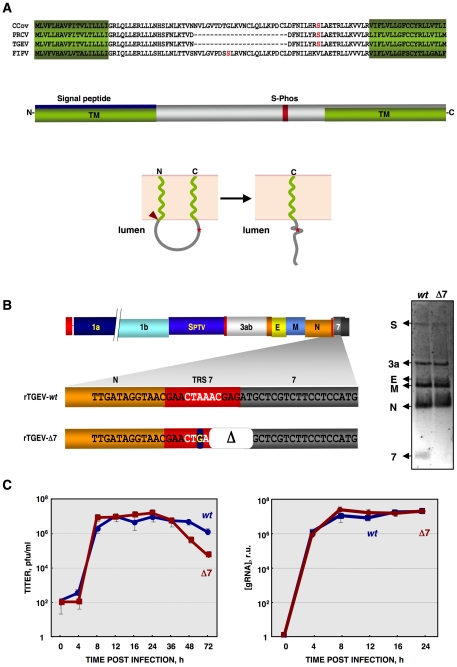
Generation of a recombinant TGEV virus lacking protein 7 expression (rTGEV-Δ7). (A) Genus α1 CoV protein 7a sequence alignment, using T-COFFEE [135]. Protein 7a sequences from the canine (CCoV) and porcine respiratory (PRCV) CoVs, transmissible gastroenteritis (TGEV) and feline infectious peritonitis (FIPV) viruses were used. GenBank accession numbers are ADB28914.1, ABG89313.1, CAA80842.1, and CAA62190.1, respectively. *In silico* prediction of TGEV protein 7 domains is represented. Transmembrane domains (TM) are in green [*PredictProtein*, [136]], the signal peptide in blue [*Signal P3.0 Server*, [137]], and a conserved phosphorilable Serine in red (S-Phos) [*NetPhos 2.0 Server*, [138]]. The predicted topology of TGEV protein 7 is also represented in lower panel [*PSORTII* [139]]. Signal peptide cleavage is indicated by a red arrowhead. S-Phos is indicated by a red star. (B) Mutations introduced to generate a rTGEV-Δ7 virus, right panel. The scheme of TGEV gRNA is shown in the upper part. The white letters represent the CS. Nucleotide change is indicated with a blue square, and the deletion (Δ) as a white square. Northern blot of subgenomic mRNAs (sgmRNAs) produced during rTGEV infections, right panel. ST cells were infected with rTGEV-*wt* (*wt*) and rTGEV-Δ7 (Δ7) viruses, at a moi of 5. Total RNA was extracted at 8 hpi. The sgmRNAs for the spike (S), 3a, envelope (E), membrane (M), nucleocapsid (N) proteins, and protein 7 were detected. (C) *In vitro* growth kinetics of the rTGEV viruses. ST cells were infected with the rTGEV-*wt* (*wt*, blue) and rTGEV-Δ7 (Δ7, red) viruses, at a moi of 5. Culture medium and total intracellular RNA were collected at different hours post infection. Intracellular RNA was only analyzed during those hours post infection in which viable cells were bound to the plate. Viral titers (left panel), and genomic RNA (gRNA) amounts (right panel), determined by RT-qPCR, were analyzed. Error bars represent the standard deviation from three independent experiments.

TGEV is a genus a1 CoV that contains three accessory genes: 3a, 3b and 7 [43], [44], [45]. The deletion of gene cluster 3ab demonstrated that these genes were not essential for *in vitro* and *in vivo* viral replication [45]. TGEV gene 7 is located at the 3′end of the genome, being the last ORF. In general, ORFs located in CoV genomes downstream of nucleocapsid (N) gene have been named as gene 7. And, one to three genes, 7a, 7b and 7c, have been described for several CoVs of genus α, β4 and γ3 at the end of their genomes [35], [46], [47] [48]. Nevertheless, most of these genes are not related to each other (J.L.G. Cruz, S. Zuñiga and L. Enjuanes, unpublished observations). In fact, new genes located in avian CoVs genomes after the N gene have been named differently as they showed no sequence homology to any other CoV genes [49]. TGEV protein 7 is similar to protein 7a of CoV genus α1, with a 72% homology to feline infectious peritonitis virus (FIPV), canine (CCoV) and porcine respiratory (PRCV) CoVs 7a proteins (Figure 1A) [50], [51]. The function of protein 7 has not been identified, and it has been proposed that it could play a role in virulence [52], [53]. The 7ab cluster deletion in FIPV (FIPV-Δ7ab) resulted in virus attenuation [54]. Nevertheless, the specific role that gene 7a plays in attenuation is not clear, as FIPV-Δ7ab phenotype was similar to the one observed for a FIPV isolate lacking only gene 7b [55].

To study gene 7 function, a recombinant TGEV virus missing gene 7 was engineered. This deletion mutant virus induced an intensified host antiviral response, including enhanced nuclease activity and eIF2α phosphorylation, leading to an increase in cell death by apoptosis. The interaction of TGEV protein 7 with PP1c was also demonstrated. Inoculation of piglets with gene 7 deletion mutant and wild-type viruses showed that virus missing gene 7 produced accelerated growth kinetics and pathology compared with that caused by the parental virus. Overall, these results indicate that TGEV gene 7 is a virulence gene that modulates host cell defenses and extends the period of virus dissemination.

## Results

### Generation of recombinant TGEV virus (rTGEV) lacking gene 7

TGEV ORF 7 encodes a 78 amino acid hydrophobic protein. The structure predicted for protein 7 contains two transmembrane domains (TM) at the amino- (aa 1–18) and carboxy-termini (aa 60–78), of the protein. The N-terminal TM domain overlaps with a signal peptide (aa 1–24) (Figure 1A). The predicted membrane topology locates the middle part of the protein towards the lumen of a membrane structure (Figure 1A). During TGEV infection, protein 7 was detected associated to the endoplasmatic reticulum (ER) and plasma membranes [56].

To study the role of protein 7 during TGEV infection, an rTGEV virus lacking gene 7 (rTGEV-Δ7) was engineered (Figure 1B) [57]. To avoid gene 7 expression, several modifications that led to an inactive ORF7 transcription regulating core sequence (CS) were introduced (Figure 1B, left panel). The two first nts of protein 7 translation start codon were also removed (Figure 1B, left panel). These mutations introduced into the TGEV infectious cDNA, were predicted to knock down gene 7 expression with minimum alteration to the 3′end of the viral genome, which is required for viral replication [58], [59]. All the mutations introduced in the cDNA were present in the recovered rTGEV-Δ7 virus, after 6 passages in tissue culture of a plaque-purified virus, indicating that they were stably maintained in the rTGEV genome. The absence of subgenomic mRNA-7 in rTGEV-Δ7 infected cells was confirmed by Northern-blot (Figure 1B, right panel). Viral titer and genomic RNA (gRNA) levels were analyzed. Intracellular RNA was only analyzed during those times post infection in which viable cells were bound to the plate (up to 24 hpi). Both mutant and parental viruses showed the same virus growth kinetics and gRNA accumulation (Figure 1C). The rTGEV-Δ7 virus titer decreased after 24 hours post infection (hpi) due to the absence of live cells. This result was expected, rTGEV-Δ7 virus titer decreased at a ratio of 1 log unit per day due to thermal instability and to the absence of viable cells, at this time post-infection, that could produce new virus [60]. These data confirmed that protein 7 was not essential for TGEV replication in cell culture.

### Cell death caused by rTGEV-Δ7 infection

**Figure 2:**
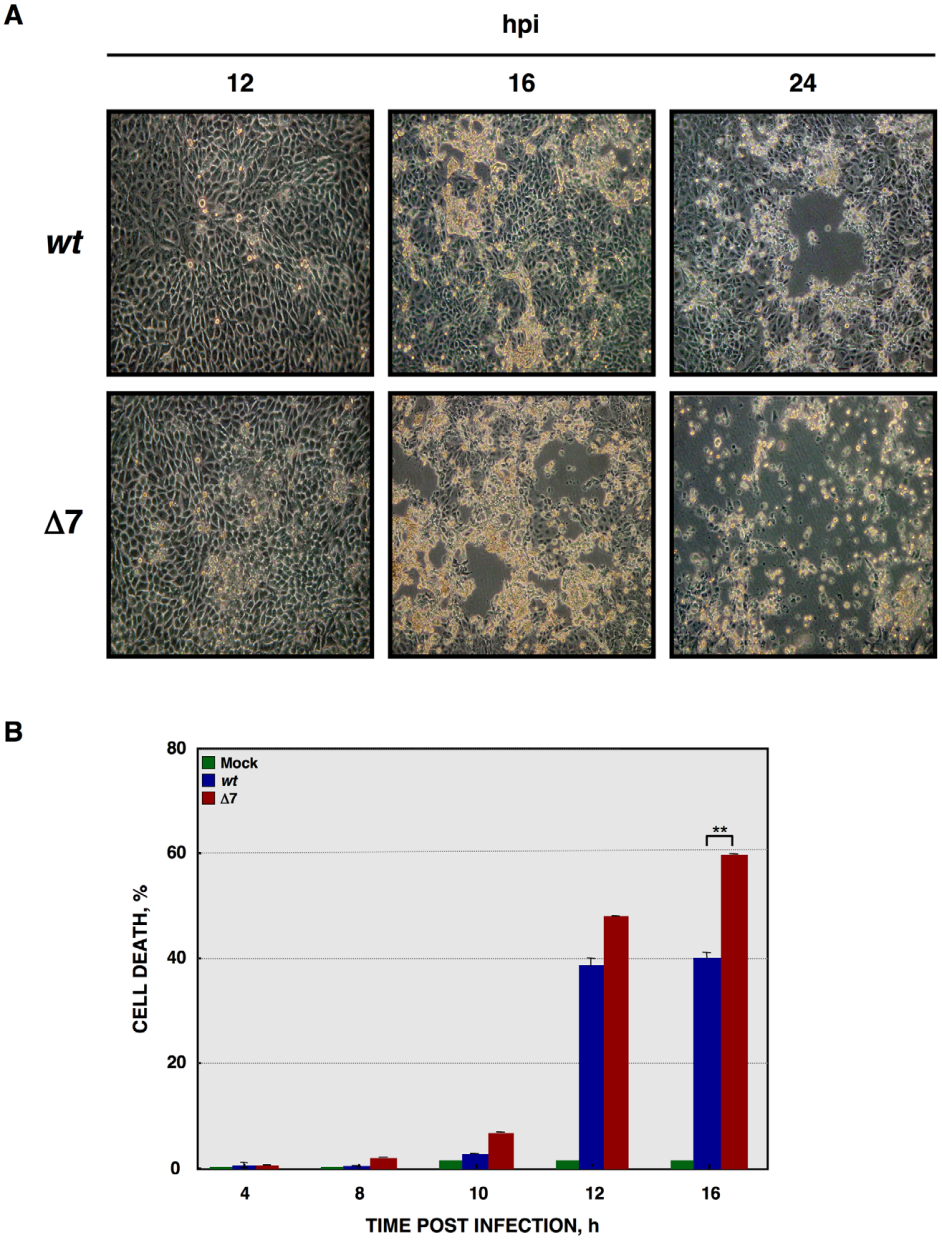
Cell death caused by rTGEV-Δ7. (A) ST cells were infected with rTGEV-*wt* and rTGEV-Δ7 (Δ7) viruses. The cytophatic effect induced by both viruses was analyzed by optical microscopy, at 12, 16 and 24 hpi. Images were taken with a 40x objective. (B) Quantification of cell death induced by rTGEV viruses. ST cells were infected with rTGEV-*wt* (*wt*) and rTGEV-Δ7 (Δ7) viruses. Cells were collected at 4, 8, 10, 12 and 16 hpi, permeabilized, and stained with propidium iodide. Dead cell population was measured by flow cytometry. Error bars indicate the standard deviation from three independent experiments. **, p-value<0.01.

The cytopathic effect (CPE), characterized by the rounding and detachment of the cells, induced by rTGEV-Δ7 virus was similar to that caused by the wild-type (rTGEV-*wt*) virus. Nevertheless, 2-fold larger plaques were produced by rTGEV-Δ7 (4 mm diameter), compared with those caused by the parental virus (2 mm diameter) (data not shown). Accordingly, in rTGEV-Δ7 infected cells the infectious foci were larger than those observed in rTGEV-*wt* infected ones at 16 hpi (Figure 2A, left panels). This increased CPE progressed until almost no viable cells remained in the rTGEV-Δ7 infection at 24 hpi (Figure 2A, right panels). The cell death induced by the rTGEV-Δ7 virus was analyzed by permeabilization, propidium iodide (PI) staining and flow cytometry (Figure 2B). This technique distinguishes dying or subdiploid cells from normal cells that emit a high PI fluorescence signal [61], [62]. As expected, the wild-type virus induced cell death and DNA degradation during the infection (Figure 2B) [63]. Interestingly, rTGEV-Δ7 caused a significant increase in cell death compared with that caused by rTGEV-*wt* infection (Figure 2B).

### Apoptosis induced by rTGEV-Δ7 virus

**Figure 3:**
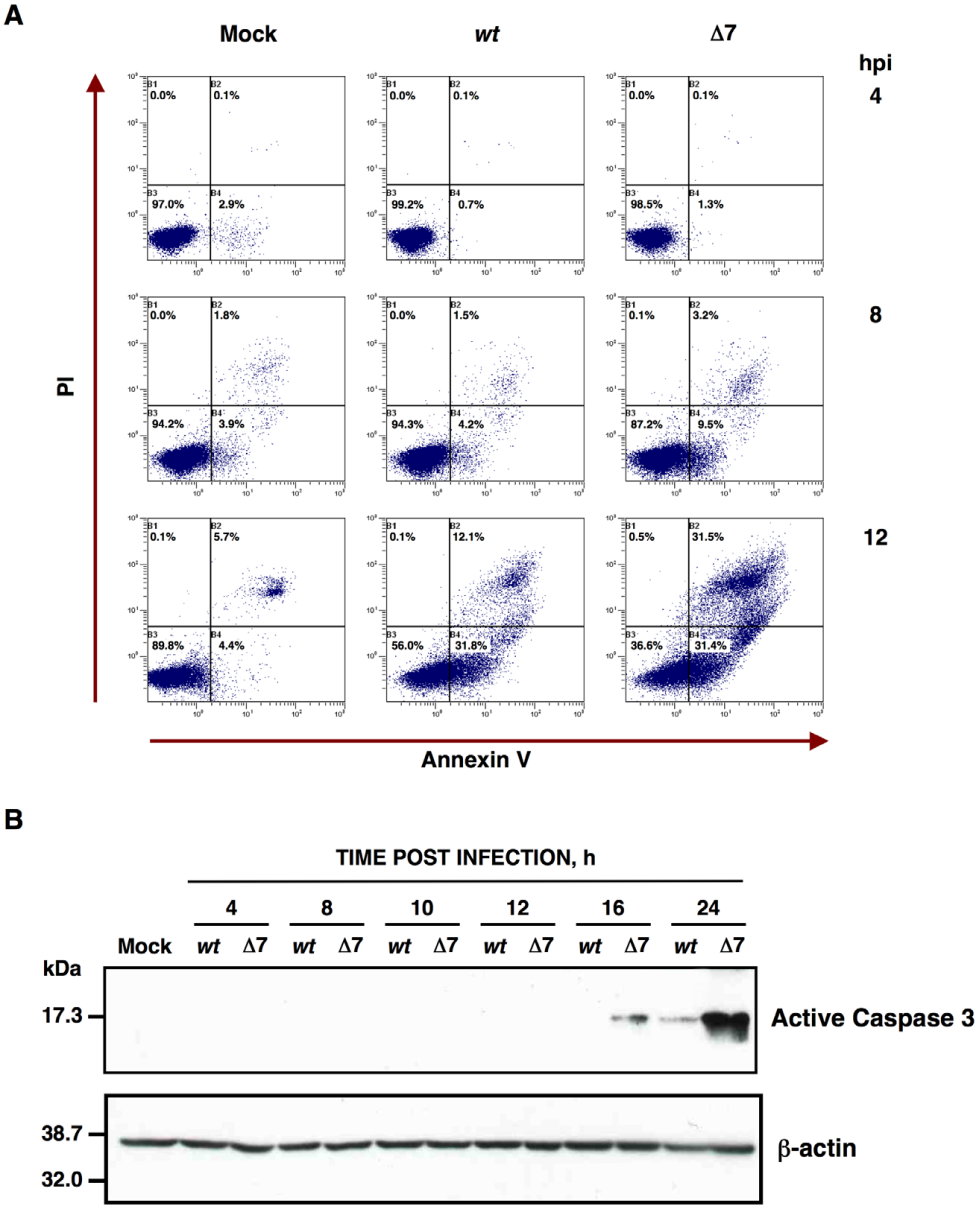
Apoptosis caused by rTGEV-Δ7. (A) Apoptosis levels in mock, rTGEV-*wt* (*wt*) and rTGEV-Δ7 (Δ7) infected cells were evaluated at 4, 8 and 12 hpi, by flow cytometry. Annexin V-PI double staining was performed to differentiate cells in early apoptosis (Annexin V^+^, PI^−^) from those in late apoptosis (Annexin V^+^, PI^+^) stages. (B) Detection of active caspase 3 by Western-blot. Total protein was extracted from ST cells infected with rTGEV-*wt* (*wt*) and rTGEV-Δ7 (Δ7) viruses, at the indicated times post infection. Active caspase 3 was detected using specific antibodies for the cleaved form. β-actin was detected as a loading control.

The main cause of the cytopathic effect induced by TGEV infection is apoptosis programmed cell death [63], [64], [65]. To analyze whether the increased cell death during rTGEV-Δ7 infection was due to an enhanced apoptosis, cells infected either with rTGEV-*wt* or rTGEV-Δ7 were simultaneously stained with PI and Annexin V, and monitored by flow cytometry. Mock infected cells remained viable (Annexin V^−^, PI^−^) throughout the experiment, indicating that the treatment did not induce apoptosis by itself (Figure 3A). As expected, the wild-type virus infection induced apoptosis (Annexin V^+^), and a cell population in late apoptosis (Annexin V^+^, PI^+^) was evident at 12 hpi (Figure 3A). Mutant rTGEV-Δ7 also triggered apoptosis but faster and stronger than that caused by the rTGEV-*wt* virus, with a 2-fold increase in apoptotic cells at 8 hpi and only 36% live cells at 12 hpi (Figure 3A).

It has previously been reported that TGEV virus induces apoptosis following a caspase dependent pathway that involves the processing of two initiator proteases (caspase 8 and 9), as well as three downstream effector caspases (caspase 3, 6 and 7) [64], [65]. Caspase 3 activation leads to TGEV N protein cleavage [64], and inhibition of caspase 3 processing, among others caspases, prevents TGEV induced apoptosis [63]. To determine the potential influence of gene 7 on caspase dependent apoptosis, the presence of the processed form of caspase 3 was analyzed by Western-blot using specific antibodies. TGEV infection induced the cleavage of caspase 3 (Figure 3B) and, as a consequence, cleaved N protein was also detected (data not shown), as expected [64]. Moreover, the rTGEV-Δ7 triggered caspase 3 processing faster than the wild-type virus. These results indicated that the increased CPE observed in rTGEV-Δ7 infected cells was most likely due to an enhanced apoptosis mediated by caspase activation.

### Effect of gene 7 deletion on macromolecular synthesis

**Figure 4:**
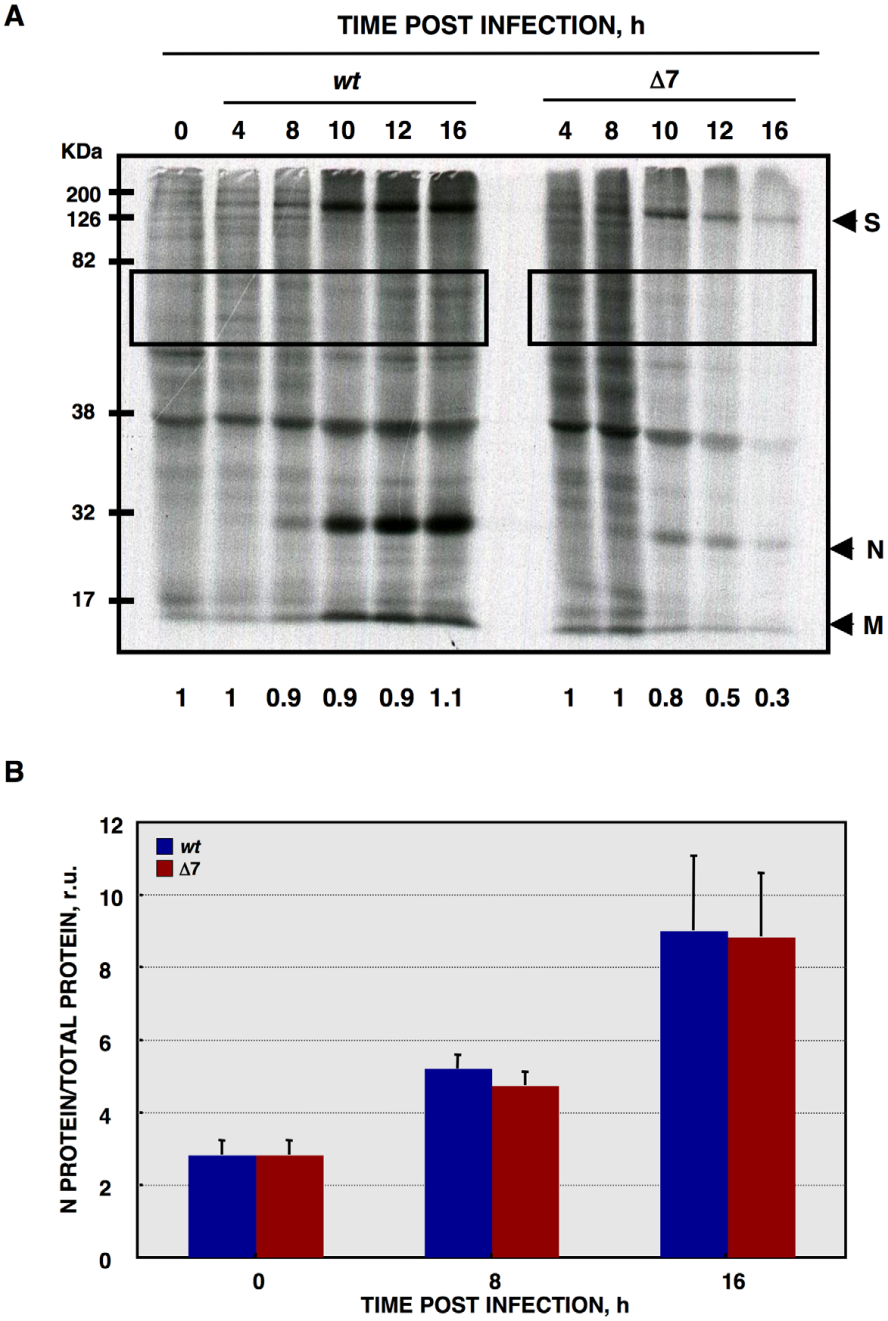
*De novo* protein synthesis in rTGEV infections. (A) At the indicated times post infection, ST cells were infected at a moi of 1 with rTGEV-*wt* (*wt*) and rTGEV-Δ7 (Δ7) viruses. Cells were labeled with^35^S Met-Cys for 30 min. Protein extracts were obtained and SDS-PAGE electrophoresis was performed to detect labeled proteins. Viral spike (S), nucleocapsid (N), and membrane (M) proteins are indicated. Densitometric analysis was performed to determine the levels of host protein synthesis. The boxes represent the region of the gel used for densitometry analysis, and the numbers below represent the relative radioactivity compared with mock-infected cells. (B) Viral-to-cell protein synthesis ratio. The amount of radiolabeled N protein, estimated by densitometry, was related to the estimated total amount of protein, at the indicated hpi. Error bars indicate the standard deviation from three independent experiments. r.u., relative units.

CoVs such as MHV or SARS-CoV, cause translational shutoff and lead to apoptosis increase [30], [66], [67], [68], [69], [70], [71]. To determine whether this was also the case for TGEV-Δ7 virus, *de novo* protein synthesis during the infection was evaluated by metabolic labeling. No translational stall was detected during rTGEV-*wt* infection (Figure 4A), as described for other CoVs such as IBV and bovine coronavirus (BCoV), or MHV at early times post infection [32], [33], [72]. In contrast, rTGEV-Δ7 infection inhibited host translational machinery, an effect detected from 10 hpi. This translational stop affected both cellular and viral protein synthesis (Figure 4A). CoVs produce viral mRNAs that are structurally similar to those produced by their host (5′ CAP-structure and poly A at the 3′end), allowing CoVs to parasitize the host translational machinery. In some CoVs, such as MHV, selective viral protein synthesis occurs concomitantly with host translational inhibition, using a mechanism not fully characterized [73], [74]. To study the mechanism responsible for protein synthesis reduction in TGEV-Δ7 infection, and to analyze whether viral mRNAs were preferentially translated, the amount of radiolabeled N protein, taken as reference for viral protein synthesis, was related to the total amount of protein (viral plus cellular) per well (Figure 4B). The ratio of viral to total protein synthesis showed no significant differences between rTGEV-*wt* and rTGEV-Δ7 infected cells (Figure 4B). In addition, no differences in viral proteins accumulation were observed at this times post infection (data not shown). These results suggested that protein synthesis at early times post infection was responsible for the virus that was still being produced after translational shutoff. This result suggested that the absence of protein 7 during TGEV infection led to protein synthesis inhibition most likely by inhibiting a cell translation step common to cellular and viral protein synthesis.

**Figure 5:**
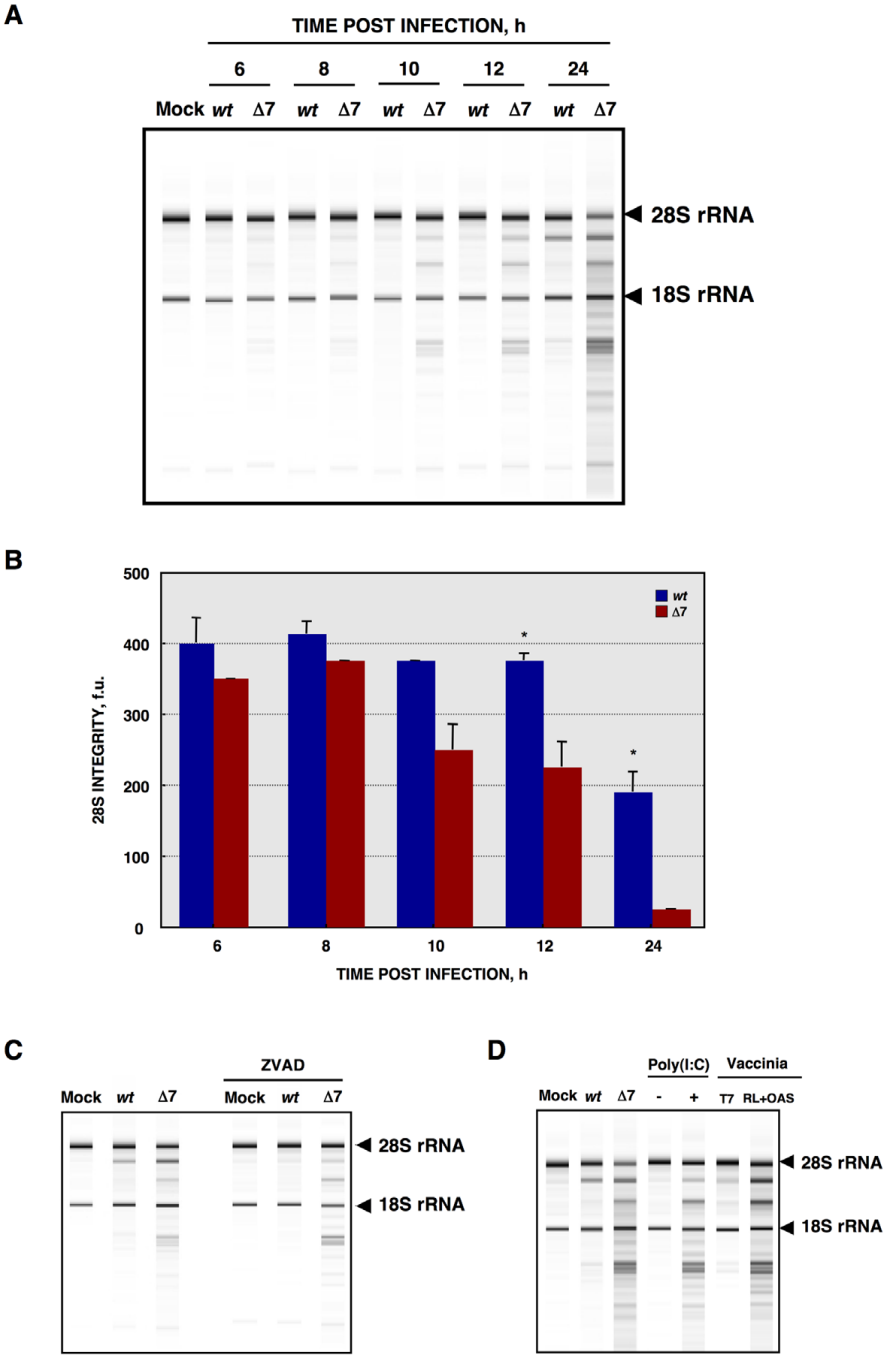
Cellular RNA integrity. (A) Total RNA extracted from infected ST cells, at indicated times post infection, was analyzed using a Bioanalyzer. The position of 28S and 18S rRNAs are indicated. (B) 28S rRNA integrity. Graph of 28S fluorescence intensity, as measured by Bioanalyzer, in the RNA samples from ST cells infected with rTGEV-*wt* (blue) or rTGEV-Δ7 (red), collected at different times post infection. f.u., fluorescence units. Error bars indicate the standard deviation from three independent experiments. *, p-value <0.05. (C) ST cells were treated with caspase inhibitor ZVAD, and infected. Total RNA was extracted and analyzed using a Bioanalyzer. (D) ST cells were transfected with Poly(I:C), and total RNA was extracted 16 hours post transfection. ST cells were also infected with a vaccinia virus expressing T7 polymerase (T7), or with the vaccinia expressing T7 polymerase, and two additional vaccinia viruses expressing 2′-5′ OAS and RNase L (RL+OAS). Total RNA was extracted 24 hpi. In all cases, cell RNA integrity was analyzed using a Bioanalyzer.

In principle, RNA decay could be responsible for the observed translational shutoff. Therefore, total cellular RNA integrity was evaluated using a Bioanalyzer [75], [76], [77]. Wild-type virus infection induced a modest RNA processing, especially at 24 hpi (Figure 5A). In contrast, rTGEV-Δ7 infection induced a faster and stronger cellular RNA degradation (Figure 5A). This data indicated that the cellular translational shutoff could be due, at least in part, to cellular mRNA degradation. Moreover, the increase in 28S rRNA degradation (Figure 5B), could affect both cellular and viral protein synthesis [78]. Nucleases activated by cell apoptosis could be responsible for the observed RNA degradation [79]. To study whether this was the case, we took advantage of the previous description of the inhibition of TGEV induced apoptosis by the addition of caspases inhibitor ZVAD, without affecting virus production [63]. In fact, after infection of ST cells with *wt* or rTGEV-Δ7 viruses in the presence of ZVAD, no CPE was observed. Total RNA was extracted from non-treated or ZVAD-treated cells, and the same RNA degradation patterns were observed in both cases (Figure 5C), indicating that the increased RNA degradation caused by rTGEV-Δ7 virus was independent of nucleases activated by cell apoptosis. To determine whether the observed cellular RNA cleavage was due to a dsRNA induced antiviral response, ST cells were treated with polyinosinic-polycytidylic acid [Poly(I:C)], which is a potent activator of this type of response [77], [80], [81]. Cells transfected with Poly(I:C) showed the same RNA degradation pattern as those infected with the rTGEV-Δ7 and parental viruses (Figure 5D), in contrast to mock treated cells. These results suggested that the cellular RNA cleavage increase, during rTGEV-Δ7 infection, was due to an enhancement of dsRNA induced antiviral activity. In general, the main effector of this process is RNase L [81], [82], [83]. To further analyze the relevance of this nuclease during TGEV infection, a recombinant vaccinia virus (VV) system was used. It was previously described that VV does not induce strong RNA degradation, due to the presence of viral genes that inhibit the RNase L system. To efficiently trigger dsRNA activated RNA degradation by RNase L, cells must be infected by VV expressing 2′-5′ OAS and RNase L [84]. Taking advantage of the wide host range of VV, porcine ST cells were infected with VV, or VVs expressing 2′-5′ OAS and RNase L. As expected, VV induced a very slight RNA degradation, that was increased by the co-expression of 2′-5′ OAS and RNase L (Figure 5D). Moreover, the RNA degradation pattern produced by the expression of RNase L system was identical to the one observed after rTGEV-Δ7 infection, strongly suggesting that RNaseL is the main nuclease involved in the increased RNA degradation after rTGEV-Δ7 infection.

**Figure 6:**
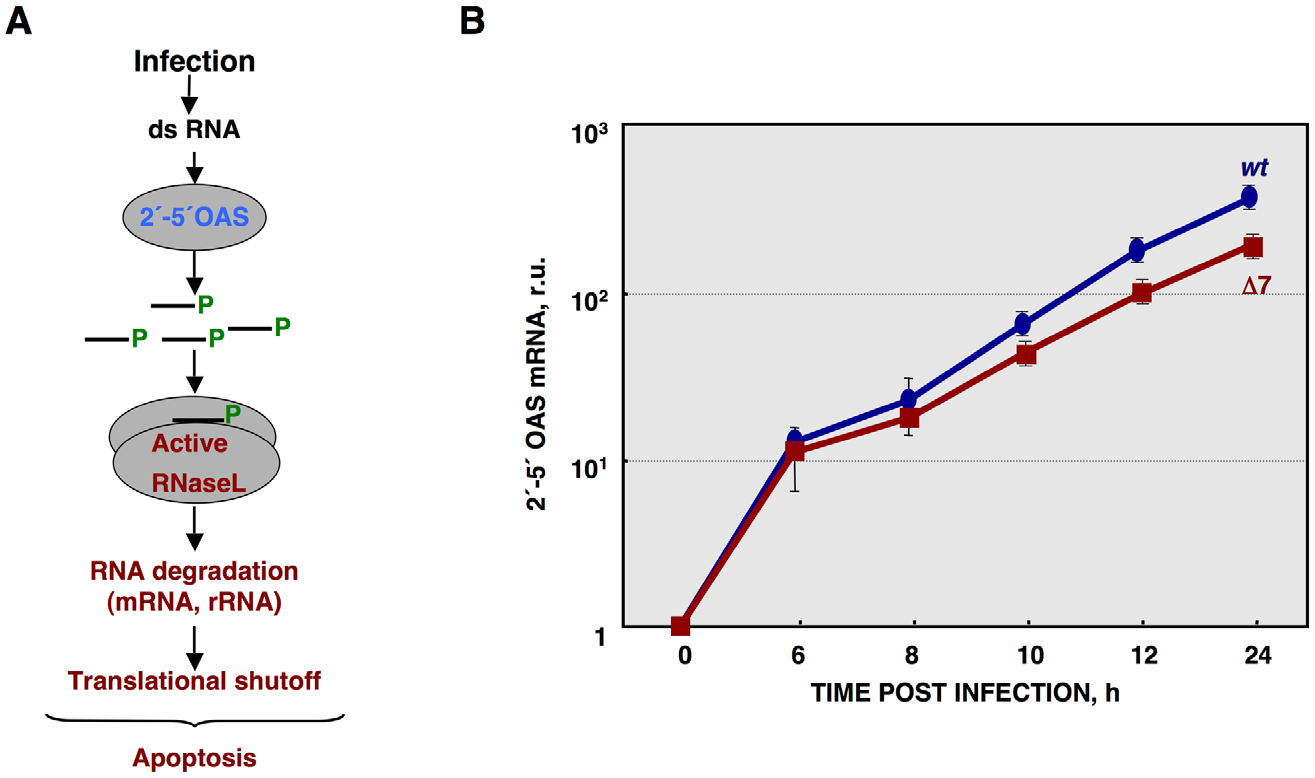
Quantification of 2′-5′OAS expression during rTGEV infection. (A) Scheme of 2′-5′OAS/RNase L activation pathway. (B) Quantification of porcine 2′-5′OAS mRNA accumulation during rTGEV-*wt* (blue) or rTGEV-Δ7 (red) infections, by RT-qPCR, at indicated time post infection. r.u., relative units. Error bars indicate the standard deviation from three independent experiments.

The activation of RNase L requires its binding to small 5′-triphosphorylated,2′-5′-oligoadenylates (2′-5′A), generated by the 2′-5′A synthetase (2′-5′OAS) [10], [11] (Figure 6A). In non-infected cells 2′-5′OAS is expressed at background levels that are significantly increased during some viral infections [85], [86]. Therefore, 2′-5′OAS1 expression during infection by rTGEV-*wt* and rTGEV-Δ7 was evaluated by quantitative RT-PCR (RT-qPCR). TGEV-*wt* infection induced the expression of the 2′-5′OAS1, as expected (Figure 6B) [87]. rTGEV-Δ7 infection also activated this pathway. Nevertheless, the slight differences in 2′-5′OAS1 gene expression between rTGEV-*wt* and rTGEV-Δ7 infections could not explain the enhanced nuclease activity observed during mutant virus infection (Figure 6B), as 2′-5′OAS1 mRNA level was even lower for rTGEV-Δ7 than for rTGEV-*wt* virus (Figure 6B).

**Figure 7:**
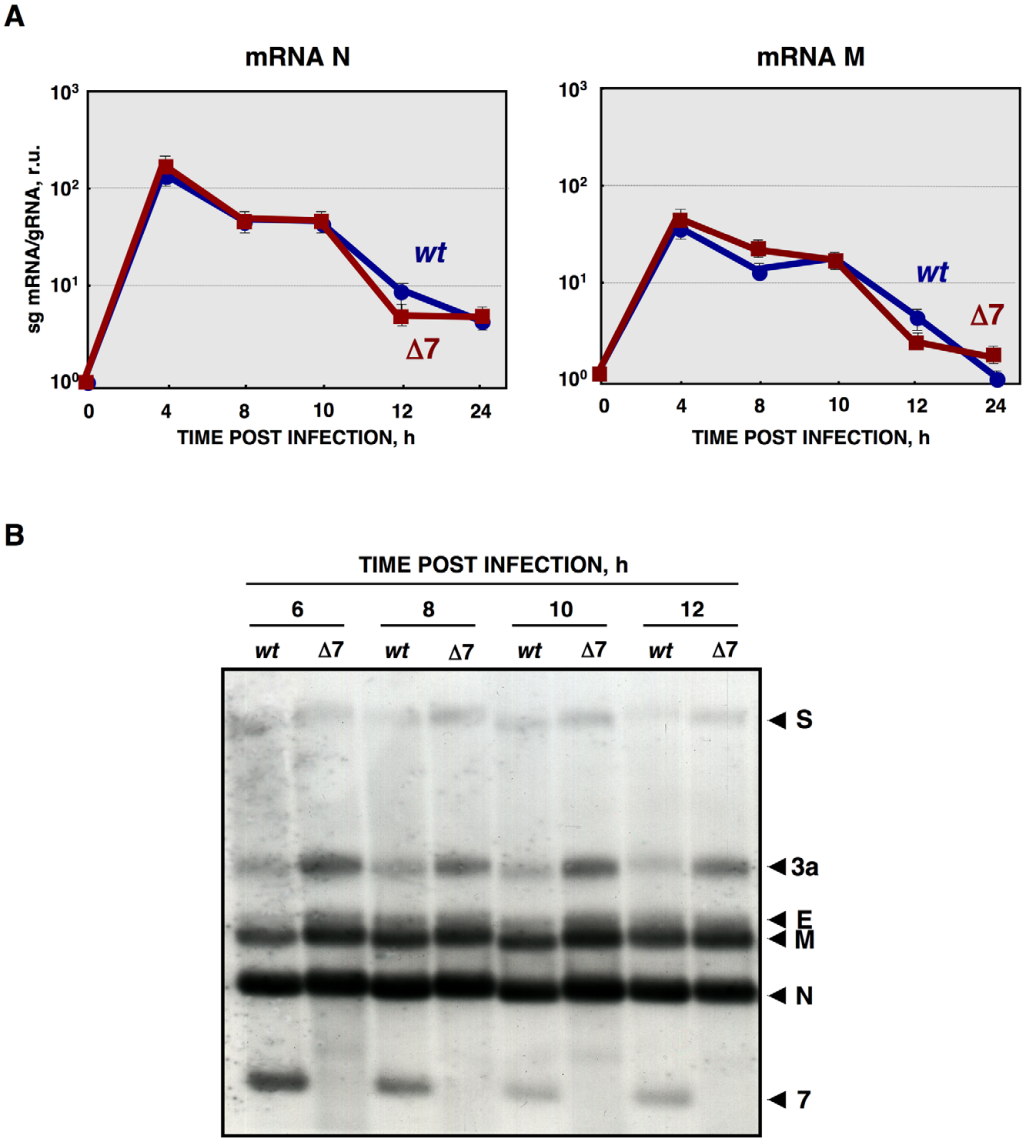
Viral RNA integrity. (A) Quantification of viral N and M sg mRNAs accumulation during rTGEV-*wt* (blue) or rTGEV-Δ7 (red) infections by RT-qPCR at indicated hpi. The ratio of sg mRNA to genomic RNA is represented. r.u., relative units. Error bars indicate the standard deviation from three independent experiments. (B) Northern blot analysis of intracellular viral sg mRNAs. ST cells were infected with rTGEV-*wt* or rTGEV-Δ7 viruses. Total RNA was extracted at indicated hours post infection and analyzed by Northern blot using a probe complementary to the 3′ end of all sg mRNAs. Total RNA amount loaded from rTGEV-Δ7 infected cells was 1.5 to 2 fold higher than that loaded from rTGEV-*wt* infected ones, in order to detect possible degradation species. Viral mRNAs for the spike (S), 3a, envelope (E), membrane (M), nucleocapsid (N) proteins, and protein 7 are indicated on the left.

Viral mRNA levels were measured by RT-qPCR, as the ratio between mRNA and gRNA amounts. No significant differences were observed between rTGEV-Δ7 and rTGEV-*wt* viruses, for the accumulation kinetics of both N and M protein mRNAs (Figure 7A). Nevertheless, RT-qPCR evaluation did not rule out whether viral rTGEV-Δ7 mRNAs could have been degraded, as the cellular RNAs were. Therefore, viral RNA integrity was evaluated by Northern blot assay. The total RNA amount loaded from rTGEV-Δ7 infected cells was 1.5 to 2 fold higher than that loaded from rTGEV-*wt* infected ones, in order to detect possible degradation species. No degradation of viral mRNAs was detected after infection by rTGEV-*wt* or rTGEV-Δ7 (Figure 7B), suggesting that viral RNAs were not degraded by the increased nuclease activity.

### Effect of protein 7 absence on translation initiation

Several mechanisms may account for the observed translational blockage. We have shown that the absence of protein 7 during TGEV infection enhanced the degradation of cellular mRNAs and ribosomal components. In addition, other factors could promote translational stall. In fact, many viruses interact with translation machinery components [88]. Eukaryotic initiation factor 4G (eIF4G) is a well-characterized target of the TGEV-induced apoptosis [64]. No difference was found in eIF4G processing at different times post infection by wild-type or mutant viruses (data not shown).

**Figure 8:**
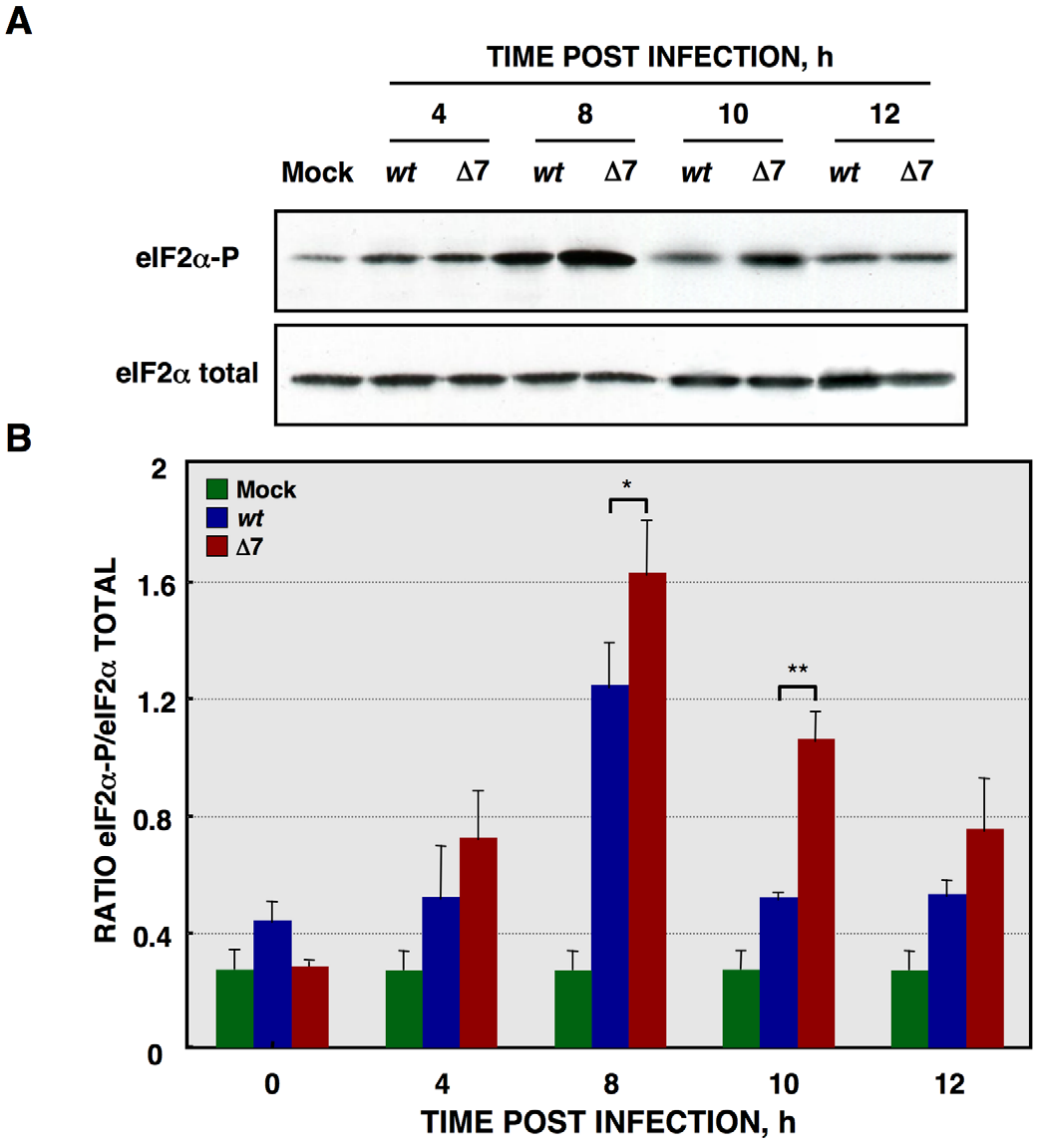
eIF2α phosphorylation during rTGEV infection. (A) Total protein was extracted, at indicated times post infection, from ST cells infected at a moi of 5 with rTGEV-*wt* (*wt*) and rTGEV-Δ7 (Δ7) viruses. Accumulation of total eIF2α and phosphorylated eIF2α (eIF2α-P), was analyzed by Western-blot. (B) eIF2α and eIF2α-P amounts were estimated by densitometric analysis. The graph represented eIF2α/eIF2α-P ratio in mock (green), rTGEV-*wt* (blue) and rTGEV-Δ7 (red) infected cells at indicated hpi. Error bars indicate the standard deviation from six independent experiments. r.u., relative units. *, p-value <0.05; **, p-value <0.01.

Protein synthesis is frequently reduced when cells are under stress, such as that caused by virus infection, by increasing the phosphorylation levels of the eIF2α subunit at serine 51 [89]. eIF2α phosphorylation, during rTGEV infection, was analyzed by Western-blot using antibodies specific for the phosphorylated (eIF2α-P) and total forms of this factor, respectively. Wild-type infection increased eIF2α-P levels (Figure 8A), reaching a maximum at 8 hpi (Figure 8B). As previously described, for other stress conditions, eIF2α-P levels decreased at late times post-infection [90], [91]. Similarly, rTGEV-Δ7 infection also induced eIF2α phosphorylation (Figure 8A) but to significantly higher levels than those observed during rTGEV-*wt* infection (Figures 8A and 8B). Interestingly, the highest difference was detected at 10 hpi, concomitantly with the time at which the mutant virus induced the translational shutoff (Figure 8B). The increased eIF2α phosphorylation was maintained, although at different levels, from 8hpi to 10 hpi, what could be sufficient to account for the translational shutoff, according to previously published studies [92], [93]. Altogether, this result indicated that, besides cellular RNA degradation, rTGEV-Δ7-induced translational shutoff is probably due to an increased and sustained eIF2α phosphorylation.

Growth arrest DNA-damage 34 (GADD34) protein is induced by cell stress, and its expression levels are upregulated on increased eIF2α phosphorylation conditions [94]. Therefore, GADD34 mRNA levels could have been modified during rTGEV-Δ7 infection, and were quantified by RT-qPCR. Infection by rTGEV-Δ7 virus induced significantly higher levels of GADD34 mRNA than the rTGEV-*wt* virus (Figure S1). This data correlated with the previous results, as higher eIF2α-P levels, in mutant virus infection, led to GADD34 increased expression.

### Protein 7 provided *in trans* restored rTGEV-*wt* phenotype

**Figure 9:**
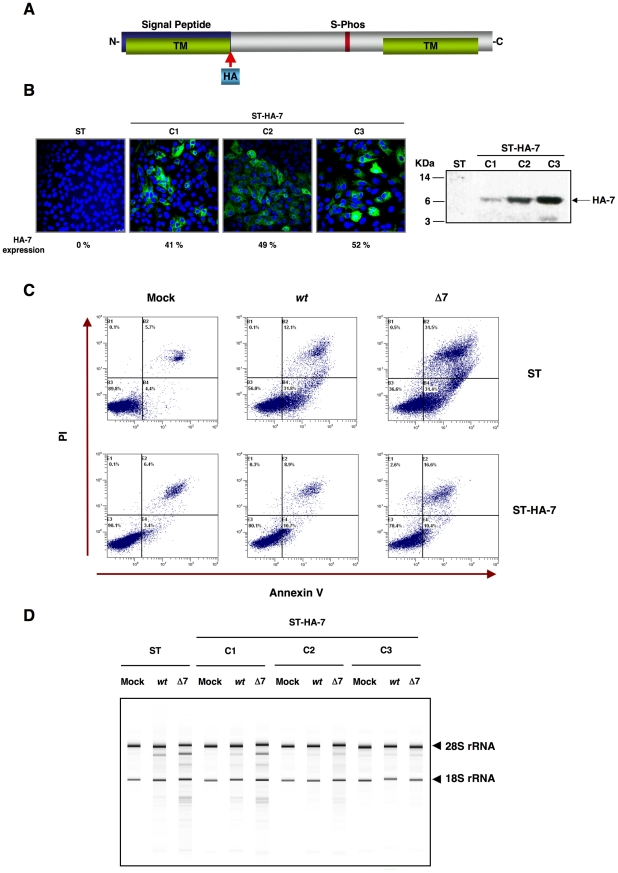
Complementation of rTGEV-Δ7 produced apoptosis and RNA degradation by protein 7 provided *in trans*. Generation of ST cells expressing TGEV protein 7 *in trans*. (A) Scheme of TGEV protein 7 expressed by the gene transfected into ST cells. Hemaglutinin tag (HA, light blue) was inserted after signal peptide (blue). (B) Protein 7 expression levels for the three ST-HA-7 selected cellular clones (C1, C2 and C3), were analyzed by immunofluorescence (left). Tagged protein 7 was detected with an anti-HA antibody stained in green, and cell nucleus were stained in blue. Percentage of HA-7 expressing cells is indicated. HA-7 protein accumulation was evaluated by Western-blot (right). HA-7 band is indicated, and corresponds to tagged protein cleaved form (7 KDa). (C) ST cells, or ST cells expressing HA-tagged protein 7 (ST-HA-7) were used to analyze apoptosis levels by flow cytometry. Apoptosis levels in mock, rTGEV-*wt* (*wt*) and rTGEV-Δ7 (Δ7) infected cells were evaluated at 12 hpi. Annexin V-PI double staining was performed to differentiate cells in early apoptosis (Annexin V^+^, PI^−^) from those in late apoptosis (Annexin V^+^, PI^+^) stages. (D) ST cells and the three ST-HA-7 cell clones obtained were mock, rTGEV-*wt* or rTGEV-Δ7 infected. Total RNA was extracted at 18 hpi. Cellular RNA integrity was analyzed using a Bioanalyzer. 28S and 18S rRNAs are indicated on the right.

To assess whether the absence of protein 7 during TGEV infection was responsible for the observed phenotype, ST cells stably expressing TGEV protein 7 (ST-HA-7) were generated. In order to detect protein 7, a hemagglutinin (HA) tag was inserted between the signal peptide and the rest of the protein (Figure 9A). Protein 7 expression was confirmed by immunofluorescence and Western-blot analysis (Figure 9B). Three ST-HA-7 cellular clones (C1, C2 and C3), with different protein 7 expression levels were selected (Figure 9B). The effect of protein 7 provided *in trans* on apoptosis and cellular RNA degradation was analyzed. Infection of ST cells by rTGEV-Δ7 caused a stronger apoptosis than the rTGEV-*wt* virus, as previously observed (Figure 9C). Protein 7 provided *in trans* significantly reduced apoptosis both in rTGEV-Δ7 infected cells and in rTGEV-*wt* infected ones (Figure 9C). Moreover, infection of ST cells by rTGEV-Δ7 caused higher RNA degradation than the rTGEV-*wt* virus, as previously described (Figure 9D). The amount of protein 7 directly correlated with the inhibition of RNA degradation, suggesting that protein 7 expression *in trans* prevented nuclease activation (Figure 9D). Furthermore, GADD34 mRNA expression (Figure S2A) and eIF2α phosphorylation levels (Figure S2B) were reduced by protein 7 expression *in trans*. Altogether, these results demonstrated that the specific phenotype of the rTGEV-Δ7 virus was due to TGEV protein 7 absence, as it was reverted to the rTGEV-*wt* phenotype, in a dose-dependent manner, by providing protein 7 *in trans*.

### Effect of protein 7 absence on the antiviral response induced by dsRNA

The activation of an antiviral response pathway triggered by the dsRNA produced during viral infections leads to eIF2α phosphorylation that results in translational shutoff [10], [95], [96]. The dsRNA-activated protein kinase (PKR) is a component of dsRNA induced antiviral response. PKR dimerization, and subsequent activation by autophosphorylation, is mediated by its binding to dsRNA [89]. Activation of PKR leads to eIF2α phosphorylation and translation inhibition (Figure S3A) [9], [12]. Infection by wild-type TGEV induced PKR phosphorylation, with a maximum at 12 hpi (Figure S3B). Nevertheless, no significant differences were observed between rTGEV-*wt* and rTGEV-Δ7 virus infections, either in PKR-phosphorylation levels or total PKR protein accumulation (Figure S3B).

During viral infection, the accumulation of nascent or misfolded proteins in the endoplasmic reticulum (ER) can trigger an ER stress pathway, which could also lead to translational stall by eIF2α phosphorylation (Figure S3A) [97]. PKR-like endoplasmic reticulum kinase (PERK) is activated by ER stress, and could participate in eIF2α phosphorylation during viral infection [98], [99]. Activation of PERK requires the prior activation of the ER chaperone immunoglobulin heavy-chain binding protein (BiP), a biomarker for the onset of the ER stress [100], [101]. Similar levels of BiP were observed in rTGEV-*wt* or in rTGEV-Δ7 infected cells during infection (Figure S3B), suggesting that PERK would not be differentially activated in the cells infected with the gene 7 deletion mutant virus with respect to those infected with the parental virus. These data strongly suggested that an increased kinase activity was not responsible for the increased eIF2α phosphorylation during rTGEV-Δ7 virus infection.

### Interaction of protein 7 and PP1

The enhanced eIF2α phosphorylation observed during rTGEV-Δ7 virus infection could be alternatively due to a decrease in the phosphatase activity that counteracts the kinases function (Figure S3A). Protein phosphatase 1 (PP1) is one of the major Ser/Thr phosphatases, and is the main enzyme responsible of the eIF2α dephosphorylation [32], [94], [102]. PP1 expression was evaluated by Western-blot, and similar protein levels were detected in both rTGEV-*wt* and rTGEV-Δ7 infected cells (Figure S3C).

**Figure 10:**
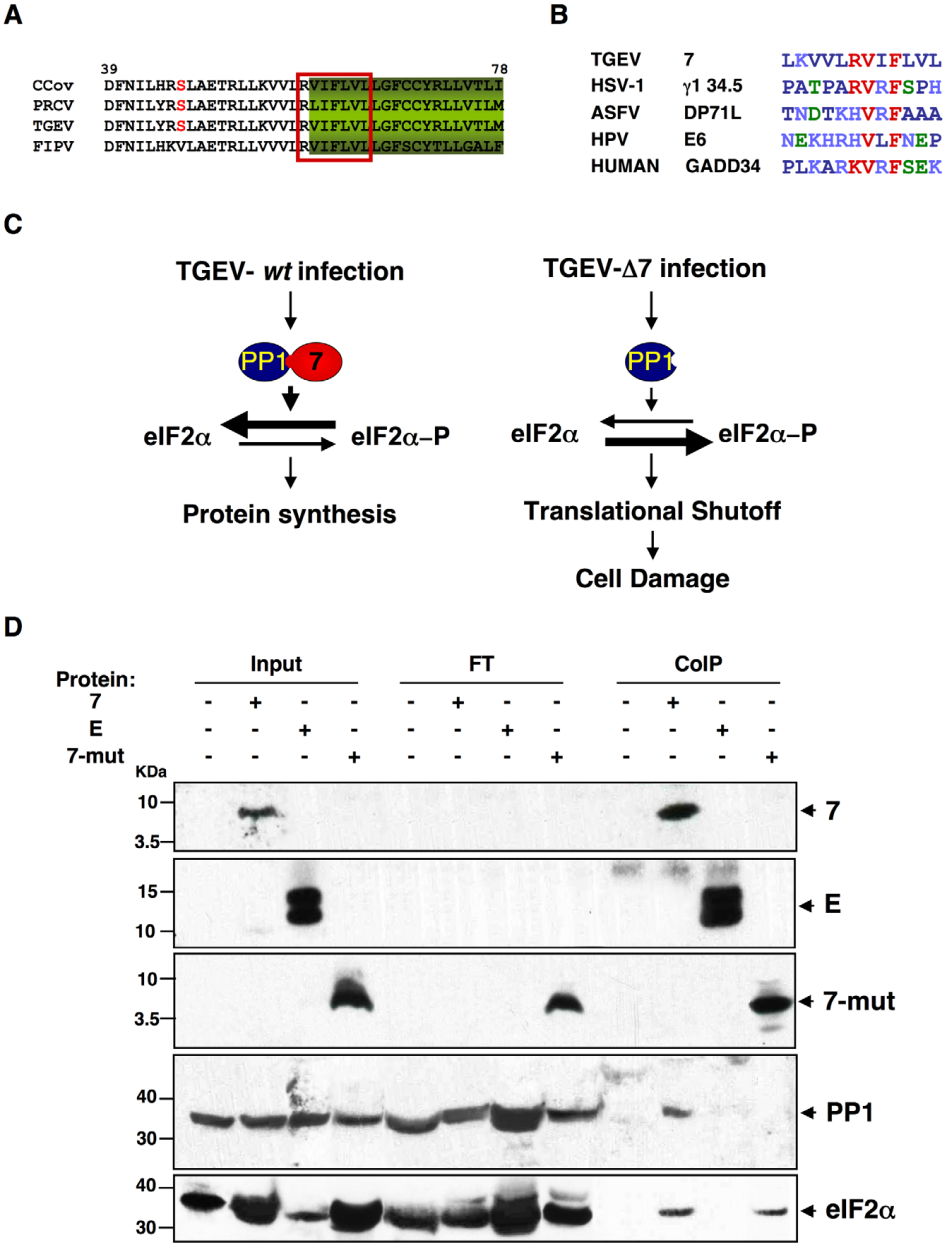
PP1c binding motif in genus α1 CoV protein 7. (A) Fragment from the alignment of genus α1 CoV 7a proteins. Canonical PP1c-binding motif is represented by the red box [ELM server [105], [106]]. (B) Consensus PP1 binding motif, including a short sequence (R/K)VxF (red), surrounded by non-polar residues. This motif is present in other viral and cellular proteins, such as human simplex virus-1 (HSV-1) γ_1_34.5, African swine fever virus (ASFV) DP71L, human papilomavirus (HPV) E6 oncoprotein, and human growth arrest DNA-damage 34 (GADD34). GenBank accession numbers are ADB28914.1, P36313, Q65212, ACR78108 and O75807, respectively. Dark blue, non-polar aa; light blue, basic aa; green, polar aa; and red, PP1 binding motif core sequence. (C) Proposed model for protein 7 function during TGEV infection. (D) Coimmunoprecipitation of TGEV protein 7 and PP1. TGEV protein 7-PP1 interaction was evaluated using ST cells, ST-HA-7 cells (7), or ST cells transiently expressing SARS-CoV E protein (E), or a protein 7 mutant lacking the PP1 binding motif (7-mut). Cell extracts were incubated with anti-HA agarose. Input, flow through (FT), and final elution (CoIP) samples were resolved by SDS-PAGE. The presence of HA-tagged proteins, PP1 and eIF2α was analyzed by Western-blot using specific antibodies.

The PP1 catalytic subunit (PP1c) can interact with more than 50 regulatory partners. The formation of these complexes determines its substrate specificity, sub-cellular location and activity, allowing PP1 to participate in numerous cellular functions [103], [104]. Therefore, although a decrease in PP1 levels was not detected in rTGEV-Δ7 infected cells, compared with rTGEV-*wt* infected ones, protein 7 could modulate PP1 activity. To study this possibility, the functional motifs of CoV genus a1 protein 7 were analyzed using the ELM server [105], [106]. A highly conserved sequence at the C-terminus of the protein was identified as the canonical PP1c-binding motif (Figure 10A). The consensus PP1c-binding motif includes a short sequence (R/K)VxF, in which x is any amino acid except those with large hydrophobic residues, surrounded by non-polar residues (Figure 10B) [103]. Previous studies have demonstrated that the RVxF motif is sufficient to mediate PP1 binding, whereas the surrounding amino acids are responsible for PP1 binding and allosteric modulation of the enzyme activity [107], [108], [109]. This motif is also present in three viral and several cell proteins, such as herpex simplex virus 1 γ_1_34.5, human papilomavirus E6 oncoprotein and African swine fever virus DP71L, and mammalian GADD34 proteins (Figure 10B). In all cases, these proteins bind PP1c and promote eIF2α dephosphorylation [94], [102], [110], [111], [112], [113]. We have observed that both rTGEV-*wt* and rTGEV-Δ7 virus infections trigger the cell antiviral response, leading to an increased eIF2α-P level. We hypothesized that during rTGEV-*wt* infection protein 7 may interact through its PP1c-binding motif with the PP1 complex, promoting eIF2α dephosphorylation, leading to normal protein synthesis (Figure 10C). In contrast, in rTGEV-Δ7 infection, the virus could not counteract the high eIF2α-P levels, causing translational shutoff and cell damage (Figure 10C). TGEV protein 7-PP1 interaction was evaluated using a pull-down assay with ST-HA-7 cells extracts. Immunoprecipitation with anti-HA-agarose followed by immunoblotting with anti-HA showed the presence of protein 7 in both the ST-HA-7 input and immunoprecipitated extracts, but not in ST cells extracts, as expected (Figure 10D). Immunoblotting with anti-PP1 confirmed that PP1c was pulled-down together with protein 7 (Figure 10D). HA-tagged SARS-CoV E protein, which is also a small viral membrane protein was used as a control bait for immunoprecipitation. The interaction between protein 7 and PP1 was specific, as E protein did not co-immunoprecipitate PP1protein (Figure 10D). Moreover, an HA-tagged protein 7 mutant, lacking PP1 binding motif, did not co-immunoprecipitate PP1 protein. Altogether, this results demonstrated TGEV protein 7-PP1 interaction. The presence of eIF2α on the co-immunoprecipitated samples was also analyzed. This factor was specifically co-immunoprecipitated both by native and mutant TGEV protein 7 (Figure 10D), suggesting that eIF2α was present in the complex formed by TGEV protein 7 and PP1. Furthermore, the interaction between protein 7 and PP1 was also evaluated in the context of TGEV infection. ST-HA-7 cells were mock infected or infected with rTGEV-Δ7, to avoid competition with the non-tagged protein 7 encoded by the wild-type virus. PP1 co-immunoprecipitated with HA-tagged protein 7 in rTGEV-Δ7 infected cells (Figure S4), indicating that TGEV protein 7 also interacts with PP1 in the context of TGEV infection. Moreover, in rTGEV-*wt* infected ST-HA-7 cells, a decrease in the PP1 co-immunoprecipitated by HA-tagged protein 7 was observed in relation to the rTGEV-Δ7 infected cells (Figure S4B), indicating that protein 7, expressed from rTGEV-*wt* virus, also interacts with PP1, and competed with tagged HA-7 protein for the binding to PP1.

**Figure 11:**
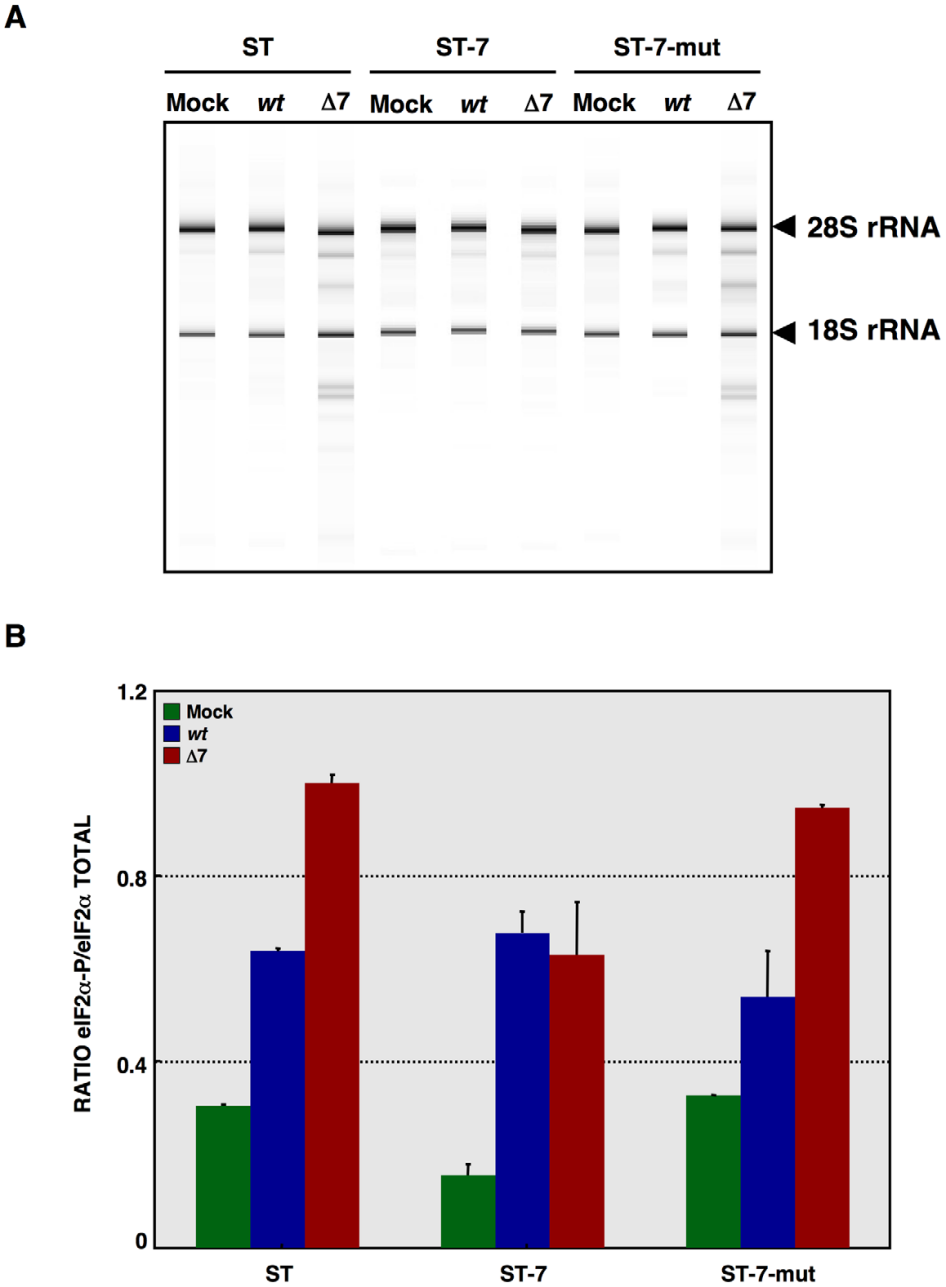
Effect of mutated protein 7 provided *in trans* on RNA degradation and eIF2α phosphorylation. ST cells, or ST cells expressing native TGEV protein 7 or the mutated protein 7 lacking PP1 binding motif were used. Cells were mock infected or rTGEV-*wt* (*wt*) and rTGEV-Δ7 (Δ7) infected. (A) Total RNA was extracted at 18 hpi and cell RNA integrity was analyzed using a Bioanalyzer. 28S and 18S rRNAs are indicated on the right. (B) Total protein was extracted at 10 hpi and eIF2α and eIF2α-P protein levels were analyzed by Western-blot. Protein amounts were estimated by densitometry, and the ratio of eIF2α-P to total eIF2α was represented. Error bars represented the standard deviation of three independent experiments.

To further evaluate the role of the PP1-protein 7 interaction on the rTGEV-Δ7 observed phenotype, RNA degradation and eIF2α phosphorylation levels were analyzed in the presence of the protein 7 mutant that did not bind to PP1. ST cells were transfected with the HA-tagged protein 7 mutant, and the expression of this protein was confirmed by immunofluorescence (data not shown). As previously observed, rTGEV-Δ7 virus caused an increased RNA degradation (Figure 11A) and eIF2α phosphorylation (Figure 11B). Interestingly, protein 7 mutant provided *in trans* did not reduce the RNA degradation and eIF2α phosphorylation caused by rTGEV-Δ7 virus (Figure 11), although eIF2α was also pulled-down by protein 7 mutant. This data strongly indicated that native TGEV protein 7 modulated RNA degradation and eIF2α phosphorylation by its interaction with PP1 protein, supporting our working hypothesis.

### *In vivo* phenotype of rTGEV-Δ7

**Figure 12:**
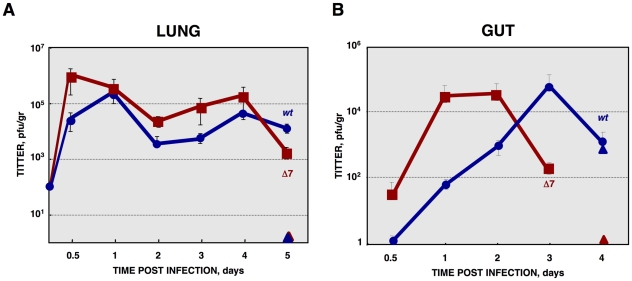
*In vivo* growth kinetics of rTGEV-Δ7 virus. (A) Two- to three-day-old piglets were inoculated with 1×10^7^ pfu/pig of rTGEV-*wt* and rTGEV-Δ7 viruses by two routes (oral and nasal) in combination. At 0.5, 1, 2, 3, 4 and 5 days post inoculation two animals per group were sacrificed, and the lungs were harvested. rTGEV-*wt* (blue) and rTGEV-Δ7 (red), recovered from lung, were titrated. Triangles indicated sentinel animals. (B) Two- to three-day-old piglets were inoculated with 1×10^7^ pfu/pig of rTGEV-SC11-*wt* and rTGEV-SC11-Δ7 viruses by three routes (oral, intranasal and intragastric) in combination. At indicated days post inoculation two animals per group were sacrificed, and the lung and the gut were harvested. rTGEV-SC11-*wt* (blue) and rTGEV-SC11-Δ7 (red) titers in gut are represented. Triangles indicate sentinel animals. Error bars indicate the standard deviation from three independent experiments.

Newborn piglets were infected with rTGEV-*wt* and rTGEV-Δ7 viruses. Both viruses showed similar growth kinetics in the lung, although gene 7 deletion mutant virus reached higher titers than the parental virus at early times post infection (Figure 12A). Virulent TGEV strains replicate in the villious epithelial cells of the small intestine and in lung cells, causing severe diarrhea in newborn piglets [57], [114], [115]. The respiratory and enteric tropism of the rTGEVs can be modified by the introduction of an S gene from a virulent strain [57], [114], [115]. The rTGEV-Δ7 deletion mutant used throughout this paper was generated with an exclusively respiratory tropism (see Materials and Methods). To study the relevance of protein 7 in a virulent virus, a recombinant virus with respiratory and enteric tropism, lacking the expression of the gene 7 (rTGEV-SC11-Δ7) was engineered [57]. Growth in lung of rTGEV-SC11-*wt* and rTGEV-SC11-Δ7 viruses was similar to that of the previous mutant and wild-type viruses (data not shown). Interestingly, the rTGEV-SC11-Δ7 showed accelerated growth kinetics in gut, compared to the wild-type virus (Figure 12B). This behavior correlated with more pronounced clinical symptoms (Figure S5A). Both rTGEV-SC11-*wt* and rTGEV-SC11-Δ7 infected animals had the same final survival ratio (50%) (Figure S5B). Nevertheless, animals infected with rTGEV-SC11-Δ7 died six days before that those infected with rTGEV-SC11-*wt* (Figure S5B). Accordingly, virus was detected only in sentinel animals in contact with rTGEV-*wt* infected piglets, but not in those in close proximity to the rTGEV-SC11-Δ7 infected animals (Figure 12B). This result suggested that the presence of protein 7 facilitated animal survival and virus shedding.

**Figure 13:**
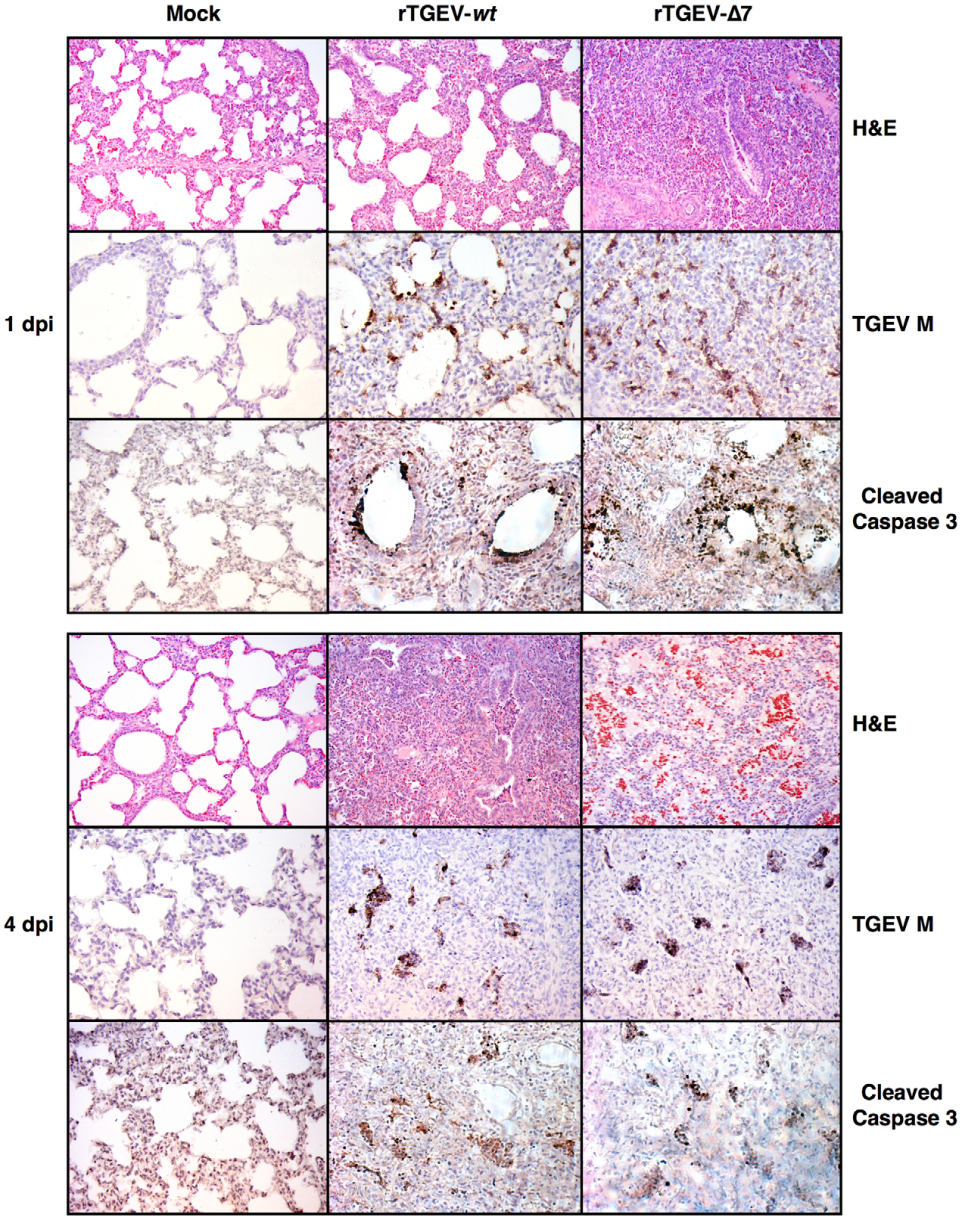
Lung histopathology caused by rTGEV-Δ7 infection. Two- to three-day-old piglets were inoculated with 1×10^7^ pfu/pig of rTGEV-*wt* and rTGEV-Δ7. Lung samples, collected at 1 and 4 days post infection, were stained with hematoxylin-eosin (H&E). Pictures were obtained with a 10x objective. TGEV membrane protein (M) and cleaved caspase 3, were also immunodetected with specific antibodies. Pictures were obtained with a 20x objective.

Histopathology of lungs from animals infected with rTGEV-*wt* and rTGEV-Δ7 viruses was analyzed. Lung injury caused by rTGEV-*wt* consisted in alveolar wall thickening, emphysemas, and obstruction of the conducting airways by cell debris (Figure 13). rTGEV-Δ7 pathology at 1dpi was comparable with that observed in piglets 4 days post rTGEV-*wt* infection, indicating that tissue injury caused by the gene 7 deletion mutant virus was faster than that due to the wild-type virus (Figure 13). In addition to the lesions described in rTGEV-*wt* infected animals, in rTGEV-Δ7 infected tissue edema was also observed as a consequence of strong alveolar congestion (Figure 13).

Virus antigen immunodetection showed the same infection pattern for both viruses (Figure 13), and the active caspase 3 pattern overlapped with those areas in which viral infectious foci were detected (Figure 13). Taken together the results indicated a faster lung infection and more extensive injury caused by rTGEV-Δ7 virus.

## Discussion

**Figure 14:**
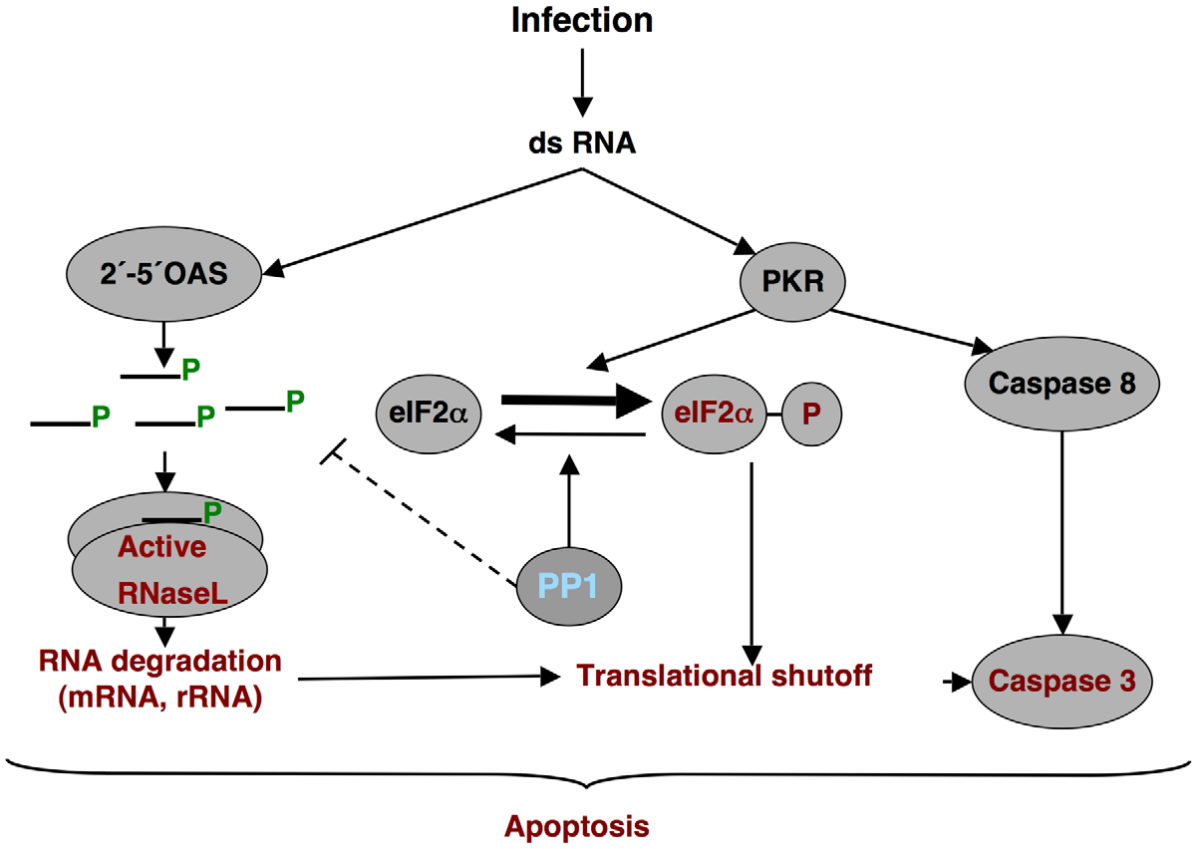
dsRNA induced antiviral pathway. Schematic overview of the dsRNA-induced antiviral pathway analyzed. Differential effects observed during rTGEV-Δ7 infection are in red. PP1, the proposed target of protein 7, is in blue.

This study shows that TGEV protein 7 modified the antiviral response, and that the presence of gene 7 attenuated virus virulence. TGEV infection led to the activation of an antiviral pathway triggered by the dsRNA produced during the virus cycle (Figure 14). This pathway has two main effectors: 2′-5′OAS that leads to RNase L activation and RNA degradation, and PKR that is responsible of eIF2α phosphorylation [116]. In general, the activation of this pathway leads to blocking of the cell translational machinery, and induction of caspase-dependent apoptosis of infected and neighboring cells (Figure 14) [8], [10]. Interestingly, we have shown that TGEV protein 7 bound PP1, a key regulator of the cell antiviral defenses, and we proposed that this binding modulates dsRNA-activated pathway.

In rTGEV-Δ7 infected cells, an increased eIF2α phosphorylation was observed over rTGEV-*wt* infection, although enhanced kinase activation was not detected. Interaction of protein 7 with the PP1c complex may counteract PKR activity (Figure 14). This is a novel mechanism not previously observed in the RNA viruses. Nevertheless, a similar mechanism was previously described for three DNA virus proteins containing a PP1c-binding motif, encoded by herpex simplex virus-1 (γ_1_34.5 protein), papilomavirus (E6 protein) and African swine fever virus (DP71L protein) (Figure 10B) [102], [110], [111]. These proteins counteract the negative effect of the eIF2α phosphorylation on cellular and viral protein synthesis through their interaction with the PP1 complex. This interaction promotes dephosphorylation of eIF2α [102], [110], [111]. In fact, while native TGEV protein 7 provided *in trans* decreased eIF2α phosphorylation, a protein 7 mutant that did not bound PP1 was unable to reduce eIF2α phosphorylation levels.

The evaluation of cellular RNA integrity in rTGEV-Δ7 infected cells revealed an increase of cellular RNA degradation compared with rTGEV-*wt* virus infected cells. The degradation pattern was identical to that observed after specific RNase L activation, suggesting that this nuclease was the responsible for RNA degradation during TGEV infection. Interestingly, 2′-5′OAS1 expression, which is required for RNase L activation, was similarly increased after infection with both rTGEV-*wt* and rTGEV-Δ7 viruses. These results suggested that protein 7 may be modulating the 2′-5′OAS pathway at a level prior to RNase L activation (Figure 14). Activation of 2′-5′OAS by dsRNA leads to the synthesis of 5′-triphosphorylated, 2′-5′-oligoadenylates (2′-5′A) required for RNase L dimerization and activation. The 2′-5′A are highly unstable due to their potential dephosphorylation at the 5′end by general phosphatases, leaving the core oligoadenylate that does not efficiently activate RNase L [117]. We propose that the complex PP1-protein 7 may counteract RNase L activation through the dephosphorylation of 2′-5′A. In fact, native TGEV protein 7 provided *in trans* reduced RNA degradation, while a protein 7 mutant that did not bound PP1 was unable to decrease RNA degradation. To our knowledge, this is the first report involving PP1 protein on the dsRNA induced RNA degradation pathway. Surprisingly, viral mRNAs were not differentially degraded after infection with rTGEV-*wt* or rTGEV-Δ7, indicating that these mRNAs may be hidden from nuclease activity. Initially, protection of these mRNAs could be mediated by their sheltering in double-membrane vesicles (DMVs), induced by CoV infection, and identified in MHV [28], SARS-CoV [29], and TGEV infected cells (A. Nogales, L. Enjuanes and F. Almazán, unpublished results). DMVs may provide an environment for viral RNA synthesis, and prevent the action of components of host defenses, such as antiviral nucleases. The mechanisms for viral mRNA protection at later stages of the viral cycle will require further studies.

We demonstrated that rTGEV-Δ7 showed an enhanced CPE, in relation to that caused by rTGEV-*wt*, which was a consequence of the acceleration of apoptosis characterized by a faster activation of caspase 3. In agreement with our results, it has previously been described that the activation of PKR and 2′-5′OAS/RNase L pathways generally leads to apoptosis [118], [119]. Furthermore, apoptosis initiated by RNase L requires caspase 3 activity [120]. Interestingly, the growth kinetics of both viruses was similar, indicating that the increased antiviral response and apoptosis, did not compromise virus replication. It has been previously described that inhibition of TGEV-induced apoptosis did not enhance viral production [63]. Similarly, in other CoVs, such as SARS-CoV or MHV, downregulation of PKR or RNase L, respectively, did not affect virus growth [96], [121]. Altogether these results suggest that, at least for these CoVs, the dsRNA-activated response did not affect viral replication. Nevertheless, all these CoVs have developed strategies to counteract the dsRNA antiviral response [33], [122], [123]. These strategies could control the deleterious effect that an exacerbated antiviral response may cause in the host, and therefore in long term virus survival [124]. In fact, rTGEV-Δ7 virus showed an accelerated growth kinetics *in vivo* compared to rTGEV-*wt*. This effect was probably due to a premature cell death in the rTGEV-Δ7 infected animal tissue that promoted a faster initial propagation of the virus.

To generate the rTGEV-Δ7 analyzed here, minimal modifications required to avoid gene 7 expression were introduced in a TGEV-wt backbone. A previously evaluated rTGEV without gene 7 expression showed full attenuation, with 100% survival of infected piglets [52], what is at variance (but not in contradiction) with the results presented in this work. Fortunately, the two deletion mutant viruses used in the Ortego et al 2003 paper and the one used here are completely different. The mutant virus in the Ortego's paper was derived from a already highly attenuated virus, only causing 20% piglet death after virus administration. It is essential to realize that this virus already included many additional attenuating genome changes: (i) five engineered restriction sites preceding genes 3a, E, M, N and 7; (ii) the duplication of sequences preceding these genes, required to avoid gene overlapping. Furthermore this duplicated sequences, located close to the gene TRS, contained an additional TRS that regulate the expression levels of each gene, what probably influenced the expression levels of these viral genes; and (iii) a deletion spanning 21 nt upstream ORF7 start codon and the first 17 nt of this ORF, that was introduced to prevent the expression of gene 7. In contrast, the TGEV deletion mutant used in this work only included a point mutation in gene 7 CS and a 7 nt deletion to prevent the production of protein 7. Therefore, the changes observed in the pathogenicity of the Ortego's recombinant virus could not be exclusively assigned to gene 7 absence, in contrast to the results presented in this paper.

In general, viral infection leads to a strong antiviral state in infected and neighboring cells [65]. We postulate that the balance between enhanced apoptosis and the bystander effect compromised and limited rTGEV-Δ7 virus tissue dissemination. Preliminary results from high throughput gene expression analysis supported this proposal (data not shown). In fact, in agreement with this postulate, rTGEV-Δ7 infected piglets showed an accelerated pathology when compared with the rTGEV-*wt* infected ones. Furthermore, the recovery from the inflammatory response was slower in rTGEV-Δ7 infected animals than in rTGEV-*wt* ones as lungs infected by the rTGEV-Δ7 showed more lesions at 4 dpi than those infected with the rTGEV-*wt*. Current work in our lab is directed at analyzing whether the removal of gene 7 in rTGEV leads to an infection with an enhanced innate immune response.

The results obtained suggested that while a balanced immune response promotes virus clearance and tissue reparation, an exacerbated innate immune response could result in immune pathology and subsequent tissue damage, as observed in rTGEV-Δ7 infected piglets. Similar effects have been described for other viruses, such as human hepatitis C virus [125], in which tissue damage was associated to the development of an exacerbated host antiviral response and not with viral replication. Moreover, piglets infected with a TGEV virulent enteric strain lacking protein 7 expression (rTGEV-SC11-Δ7), developed a faster and more pronounced clinical disease. High pathogenicity resulted in a more rapid host elimination, affecting virus long-term survival as the host is essential for virus propagation. From an evolutionary point of view, our results suggested that CoVs genus α1 might have acquired gene 7 to counteract host defenses with the aim of preventing overwhelming tissue damage due to an exacerbated innate immune response. Protein 7 would then benefit both the host, reducing the pathology caused by the infection, and the virus, allowing longer virus persistence and dissemination.

## Materials and Methods

### Ethics statement

Animal experimental protocols were in strict accordance with EU guidelines 2010/63/UE, and Spain national law RD 1201/2005, about protection of animals used for experimentation and other scientific purposes, and national law 32/2007, about animal welfare in their exploitation, transport, experimentation and sacrifice. The experiments were performed in an animal facility at Pfizer Animal Health, Girona (Permit numbers G9900005 and G9900007), and were approved by the in site ethical review committee (Comitè Ètic d'Experimentació Animal).

### Cells

Baby hamster kidney (BHK) cells stably transformed with the porcine amino peptidase N gene (BHK-pAPN) [126] were grown in Dulbecco's modified medium (DMEM) supplemented with 5% fetal bovine serum (FBS) and G418 (1.5 mg/ml) as a selection agent. Swine testis (ST) cells were grown in DMEM supplemented with 10% FBS [127].

### Generation of ST cells expressing TGEV protein 7

The gene for TGEV protein 7, with hemaglutinin tag (HA) inserted after the signal peptide, cloned in *Hind*III*-Eco*RI restrictions sites in the plasmid pcDNA 3.1, was purchased from GenArt (Germany). Four micrograms of pcDNA 3.1-HA-7 were linearized with *Sma*I, and purified using QIAquick Kit (Qiagen) according to the manufactureŕs specifications. The linearized plasmid was used for reverse transfection of ST cells with 12 µl of Lipofectamine 2000 (Invitrogen), as recommended by the manufacturer. Cells were grown in DMEM supplemented with 10% FBS and G418 (1.5 mg/ml) as a selection agent. Cells were cloned and positive clones for HA-7 expression, by immunofluoresce and Western-blot, were amplified.

### Generation of ST cells transiently expressing TGEV protein 7-mut or SARS-CoV E protein

A pcDNA 3.1 plasmids, with TGEV 7-mut gene cloned in *Hind*III*-Eco*RI restrictions sites, was purchased from GenArt (Germany). This plasmid, pcDNA3.1-7-mut, encodes TGEV protein 7 with a deletion comprising amino acids 59 to 62, which include the PP1 binding motif (R/K)VxF, with an HA tag inserted after the signal peptide. Plasmid pcDNA3.1-E, encoding SARS-CoV E protein, with an HA tag in its amino-terminus, was previously obtained in our laboratory (E. Alvarez, M. L. DeDiego and L. Enjuanes, unpublished results). For transient expression experiments, circular plasmids were used for reverse transfection of ST cells as described above.

### Construction of the plasmid pBAC-TGEV-Δ7

A recombinant TGEV virus was engineered using a TGEV-SPTV genetic background, with respiratory tropism and adapted to tissue cultures [57]. The mutations required to knock down gene 7 expression were introduced by overlapping PCR using as a template the plasmid pSL-3EMN7, comprising nucleotides 20,372 to 28,087 of TGEV genome [128]. Overlapping PCR fragments, with point mutations and deletions, were amplified using oligonucleotides ΔORF7 VS (5′-GCTCGTCTTCCTCCATGCTGTATTTAT-3′) and ΔORF7 RS (5′-GATAATTGATGAGGTAACGAACTGAGCTCGTCTTCGTTACCTATC-3′).

The final PCR product (2700 bp), amplified with outer oligonucleotides ΔORF7 VS-Oli 4 *Sph*I RS (5′-CATAGCACAATAGCGTTCTCCACATGCGCATGCA-3′) and ΔORF7 RS-Oli 1 *Sph*I VS (5′-GGAGGATTGGGAAGACAATAGCAGGCATGCTGGGG-3′), was digested with *Sph*I and cloned in the same restriction site of pSL-3EMN7, leading to pSL-3EMNΔ7. To generate the plasmid pBAC-TGEV-SPTV-Δ7, pSL-3EMNΔ7 was digested with *Sfo*I-*Bam*HI. This fragment, containing nt 23,464 to 28,700 of the TGEV genome, and including the mutations, was cloned in the same restriction sites of the full-length pBAC-TGEV-SPTV^FL^ [129]. To generate a rTGEV-Δ7 virus with both enteric and respiratory tropism a TGEV-SC11 virus backbone was used [57]. To this end, the pSL-3EMNΔ7 *Sfo*I-*Bam*HI fragment was cloned in the same restriction sites of the full-length pBAC-TGEV-SC11^FL^ [129]. All cloning steps were checked by sequencing of the PCR fragments and cloning junctions.

### Transfection and recovery of infectious virus

BHK-pAPN cells were grown to 95% confluence on 35-mm-diameter plates and transfected with 4 µg of infectious cDNA using 12 µl of Lipofectamine 2000 (Invitrogen), according to the manufacturer's specifications. After 6 h of incubation at 37°C, cells were trypsinized and plated over a confluent ST monolayer grown in 35-mm-diameter plate. Recombinant TGEV (rTGEV) viruses were recovered, grown and titrated as previously described [130], [131].

### RNA extraction and analysis

One day after confluence ST cells, grown on 35-mm-diameters plates, were infected at a multiplicity of infection (moi) of 5. Total intracellular RNA was extracted at different hours post-infection (hpi) using the RNeasy Mini Kit (Qiagen), according to the manufacturer's instructions. Viral sg mRNAs were evaluated by Northern blot and RT-qPCR analyses, following standard procedures set up in our laboratory [131], [132]. Cellular gene expression was analyzed by using a custom TaqMan gene expression assay (Applied Biosystems) specific for porcine 2′,5′oligoadenylate synthetase 1 (2′,5′OAS1) (Table 1), and growth arrest DNA-damage 34 (GADD34) (Table 1). Data were acquired with an ABI PRISM 7000 sequence detection system and analyzed with ABI PRISM 7000 SDS version 1.2.3 software (Applied Biosystems). Total cell RNA integrity was evaluated with a Bioanalyzer 2100 (Agilent Technologies) following the manufacturer̀s recommendations, and analyzed with 2100 Expert software (Agilent Technologies). Four micrograms of polyinosynic-polycytidylic acid [Poly (I:C), Sigma] were used for reverse transfection of ST cells with 12 µg of Lipofectamine 2000 (Invitrogen), as recommended by the manufacturer. Total RNA was extracted 16 hours post transfection, and cell RNA integrity was analyzed as described above. For apoptosis inhibition experiments, caspase inhibitor inhibitor N-benzyloxycarbonyl-Val-Ala-Asp-fluoromethylketone (ZVAD.fmk) was added to the cell culture medium at a concentration of 100 µM as previously described [63]. Total RNA was extracted 18 hours post infection, and cell RNA integrity was analyzed as described above.

**Table 1:** Accession numbers of proteins mentioned in the text.

PROTEIN	SPECIES (a)	ID	DATABASE
β-actin	Porcine	Q7M3B0	UniProtKB (unreviewed)
	Human	P60709	UniProtKB
BiP	Porcine	P34935	UniProtKB
	Human	P11021	UniProtKB
Caspase 3	Porcine	Q95ND5	UniProtKB
eIF2α	Porcine	P20460 (b)	UniProtKB
	Human	Q9BY44	UniProtKB
eIF4G	Porcine	– (c)	–
	Human	Q04637	UniProtKB
GADD34	Porcine	ENSSSCT00000003504	ENSEMBL
OAS1	Porcine	Q29599	UniProtKB
PERK	Porcine	ENSSSCP00000008763	ENSEMBL
	Human	Q9NZJ5	UniProtKB
PKR	Porcine	Q865A4	UniProtKB (unreviewed)
	Human	P19525	UniProtKB
PP1	Porcine	–	–
	Human	P62136	UniProtKB
RNaseL	Porcine	A5H025	UniProtKB (unreviewed)
	Human	Q05823	UniProtKB

(a) The work was performed in porcine cells, but human IDs are also provided when antibodies for human proteins were used.

(b) Available sequence corresponds to a 70 aa fragment.

(c) Sequence not available.

### Expression of RNase L system from recombinant vaccinia viruses

To evaluate the cellular RNA degradation by the 2-5OAS/RNase L system, three recombinant vaccinia viruses, vvT7, vvRL and vv2-5AS, were used as previously described [84]. Expression of RNase L from vvRL, was under the control of T7 promoter [133]. Expression of T7 polymerase and human 2-5OAS1, produced by vvT7 and vv-2-5AS respectively, was constitutive. ST cells were infected at a moi of 2 with vvT7 or vvT7, vvRL and vv2-5AS. Total RNA was harvested at 24 hpi, and analyzed by a Bioanalyzer as described above.

### Protein analysis by Western blotting

ST cells were infected at a moi of 5, harvested at different hpi, and protein extracts were obtained as previously described [59]. When protein phosphorylation levels were analyzed, a phosphatase inhibitor cocktail (PhosSTOP, Roche) was added to the extraction buffer. Cell lysates were separated by sodium dodecyl sulfate-polyacrylamide gel electrophoresis (SDS-PAGE). Proteins were transferred to a nitrocellulose membrane (Hybond-C, GE Healthcare) and analyzed as described [45]. The membranes were incubated with polyclonal antibodies (pAbs) specific for active Caspase 3 protein (abcam, 1∶10,000), PKR (Santa Cruz, 1∶200), BiP (Abcam, 1∶500), eIF2α (Santa Cruz, 1∶2000), phosphorylated eIF2α (Invitrogen, 1∶500) and PP1c (Santa Cruz, 1∶200). Monoclonal antibodies (mAbs) specific for HA (Sigma, 1∶1000), total PKR (Santa Cruz, 1∶1000), PP1c (Santa Cruz, 1∶1000) and β-Actin (Abcam, 1∶10,000) were also used. Protein accession numbers are detailed in Table 1. Bound primary antibodies were detected with horseradish peroxidase-conjugated antibodies specific for the different species, using the Immobilon Western chemiluminescent substrate (Millipore), following the manufactureŕs recommendations. Protein amounts were estimated by densitometric analysis using Quantity One 4.6.3 software (BioRad). At least three different experiments and appropriate gel exposures were used in all cases with similar results. In addition, different exposures of the same experiment were analyzed to assure that data were obtained from films within linear range.

### Immunofluorescence

ST-HA-7 cells were fixed with 4% paraformaldehyde and permeabilized with 0.2% saponin in phosphate-buffered saline (PBS) and 10% FBS. Monoclonal antibody specific for HA (Sigma, 1∶500) was used. Bound primary antibody was detected with AlexaFluor488 conjugated antibody specific for mouse (Invitrogen, 1∶500). Cell nucleus were stained with 4′,6-diamidino-2-phenylindole (DAPI) (Sigma, 1∶200).

### Metabolic labeling

One day post-confluence ST cells, grown on 35-mm-diameters plates were infected at a moi of 1 to avoid strong cytopathic effect (CPE). The cells were incubated 30 min in cysteine- and methionine- free modified Eaglés medium with 10% FBS (starvation medium). The medium was then replaced by starvation medium containing 50 µCi ^35^S/ml labeled Met and Cys (Taper). Cells were incubated at 37°C for 1 hour, washed with PBS containing 50 mM Ca^2+^ and 50 mM Mg^2+^, and pelleted. The cells were broken in 50 µl of lysis buffer [59] supplemented with a nuclease mix (10U DNaseI from Roche, 10 µg RNase A from Qiagen) and 50 µl of SDS-PAGE loading buffer 2x [134]. Total protein lysates were subjected to one freeze-thaw cycle and then boiled at 95°C for 10 min, 15 µl of each sample were separated by 5-15% gradient SDS-PAGE. The gel was dried under vacuum onto Whatman 3 MM paper and exposed for protein product visualization. Label was estimated by densitometric analysis as described above.

### Cell death analysis

To quantify cell death levels, ST cells were permeabilized and stained with vital dye propidium iodide (PI) (Roche) following standard procedures (Nicoletti I., 1991). The cell death population (genomic content <2 n) was quantified by flow cytometry. Apoptosis was evaluated by flow cytometry using fluorescein isothiocyanate (FITC) conjugated Annexin V (Roche), specifically binding apoptotic cells, as previously described [61]. Annexin V plus PI double staining was performed to differentiate cells in early apoptosis (Annexin V^+^, PI^−^) from those in late apoptosis (Annexin V^+^, PI^+^) stage.

### Co-immunoprecipitation

Cell extracts from ST-HA-7 cells, expressing TGEV HA-tagged protein 7, or ST cells transiently expressing HA-tagged coronavirus proteins 7-mut or E were incubated with a mAb anti-HA agarose conjugated (1∶1, Sigma), following the manufactureŕs recommendations. The presence of viral proteins 7, 7-mut and E, and cell proteins PP1 and eIF2α in the eluted samples was analyzed by Western-blot using specific antibodies as described above.

### *In vivo* growth kinetics

Two- to three-day-old non-colostrum-deprived piglets, born from TGEV seronegative sows, were inoculated with virus (1×10^7^ pfu/pig) following standard procedures [57]. Briefly, for respiratory tropism viruses animals were infected by two different routes (oral and intranasal) in combination. For enteric tropism viruses, animals were infected by three routes (intranasal, oral and intragastric) in combination. Infected animals were monitored daily to detect symptoms of disease and death. At 0.5, 1, 2, 3, 4 and 5 days post-inoculation (dpi) two animals per group were sacrificed, and the lungs were collected. In order to evaluate representative samples, tissue extracts were obtained by homogenizing the whole organs at 4°C by using a Pro-250 tissue homogenizer (Fisher Scientific). Virus titers were determined in lung extracts following procedures set up in the laboratory [57].

### Immunohistochemistry

Lung representative sections were fixed with 4% paraformaldehyde and stored in 70% ethanol at 4°C. Paraffin embedding, sectioning and hematoxylin-eosin staining (H&E) were performed by the histology service in the National Center of Biotechnology (CNB, Spain). 4 micron sections were immunostained for TGEV membrane (M) protein and cleaved caspase 3. Briefly, samples were deparaffined at 60°C and rehydrated by successive incubations in 100% xylol, 100% ethanol and 96% ethanol. Endogenous peroxidase was blocked at 37°C in darkness with 1% H_2_O_2_ diluted in methanol. For cleaved caspase 3 detection, tissue sections were boiled in citrate buffer (8.2 mM sodium citrate; 1.8 mM citric acid) pH 6.5. Unspecific binding was blocked with 3% bovine serum albumin (BSA) in PBS. Samples were incubated with a mAb specific for TGEV M protein (3B.B3, 1∶100) [130] or with a pAb specific for active caspase 3 protein (abcam, 1∶300), respectively. Bound primary antibodies were detected with biotinylated antibodies specific for the different species, using the ABC Peroxidase Staining Kit and Metal Enhanced DAB Substrate Kit (Pierce), following the manufactureŕs recommendations.

## Supporting Information

Figure S1 Porcine GADD34 expression. The expression of porcine GADD34, during rTGEV-*wt* (blue) or rTGEV-Δ7 (red) infections at indicated hpi, was analyzed by RT-qPCR. Error bars indicate the standard deviation from three independent experiments. r.u., relative units.
(TIF)

Figure S2 Decreased eIF2α-P by expression of TGEV protein 7 *in trans*. (A) ST cells and ST-HA-7 clones C1, C2 and C3 were infected with rTGEV-*wt* or rTGEV-Δ7. Total RNA was extracted at 10 hpi and porcine GADD34 expression was analyzed by RT-qPCR. r.u., relative units. Error bars represented the standard deviation of three independent experiments. (B) ST cells and ST-HA-7 clones C1 and C3 were infected with rTGEV-*wt* or rTGEV-Δ7. Total protein was extracted at 10 hpi and eIF2α and eIF2α-P protein levels were analyzed by Western-blot. Protein amounts were estimated by densitometry, and the ratio of eIF2α-P to total eIF2α was represented. Error bars represented the standard deviation of three independent experiments.
(TIF)

Figure S3 Effect of protein 7 on kinases implicated in eIF2α phosphorylation. (A) Scheme of eIF2α/eIF2α-P equilibrium influenced by PKR, PERK and PP1 activity. (B) Evaluation of phosphorylated PKR (PKR-P), total PKR and BiP accumulation during rTGEV-*wt* or rTGEV-Δ7 infections, at indicated hpi, by Western-blot using specific antibodies. β-actin was detected as loading control. (C) Analysis of PP1 accumulation in ST cells infected with rTGEV-*wt* or rTGEV-Δ7 at indicated times post infection, by Western-blot using a specific antibody. β-actin was detected as loading control.
(TIF)

Figure S4 Interaction between PP1 and TGEV protein 7 in the context of TGEV infection. (A) ST mock infected cells, or ST-HA-7 cells infected with rTGEV-Δ7 were used for immunoprecipitation. Cell extracts from 16 hpi were incubated with anti-HA agarose. Input, flow through (FT), and final elution (CoIP) samples were resolved by SDS-PAGE. The presence of HA-tagged protein 7 and PP1 was analyzed by Western-blot using specific antibodies. (B) ST-HA-7 mock infected cells, or infected with rTGEV-*wt* or rTGEV-Δ7 viruses were used for immunoprecipitation as in (A). Co-immunoprecipitated (Co-IP) samples from different experiments were resolved by SDS-PAGE, and HA-tagged protein 7 and PP1 were detected by Western-blot. The graph represents the ratio between PP1 and HA-7 protein, estimated by densitometry. Error bars represent the standard deviation from the different experiments.
(TIF)

Figure S5 *In vivo* rTGEV-SC11-Δ7 virulence. Three-day-old piglets were inoculated with 1×10^7^ pfu/animal of rTGEV-SC11-*wt* or rTGEV-SC11-Δ7 viruses, by three routes (oral, intranasal and intragastric) in combination. (A) Clinical symptoms were analyzed during the experiment. The degree of diarrhea was represented: from 0, meaning healthy animal, to 3, meaning acute diarrhea. (B) Number of surviving piglets at different days post inoculation.
(TIF)
