# Supplementary figures and images for: Coronavirus Gene 7 Counteracts Host Defenses and Modulates Virus Virulence
Source: PLoS Pathog. 2011 Jun 9;7(6):e1002090. doi: 10.1371/journal.ppat.1002090 (PMC3111541; doi:10.1371/journal.ppat.1002090)

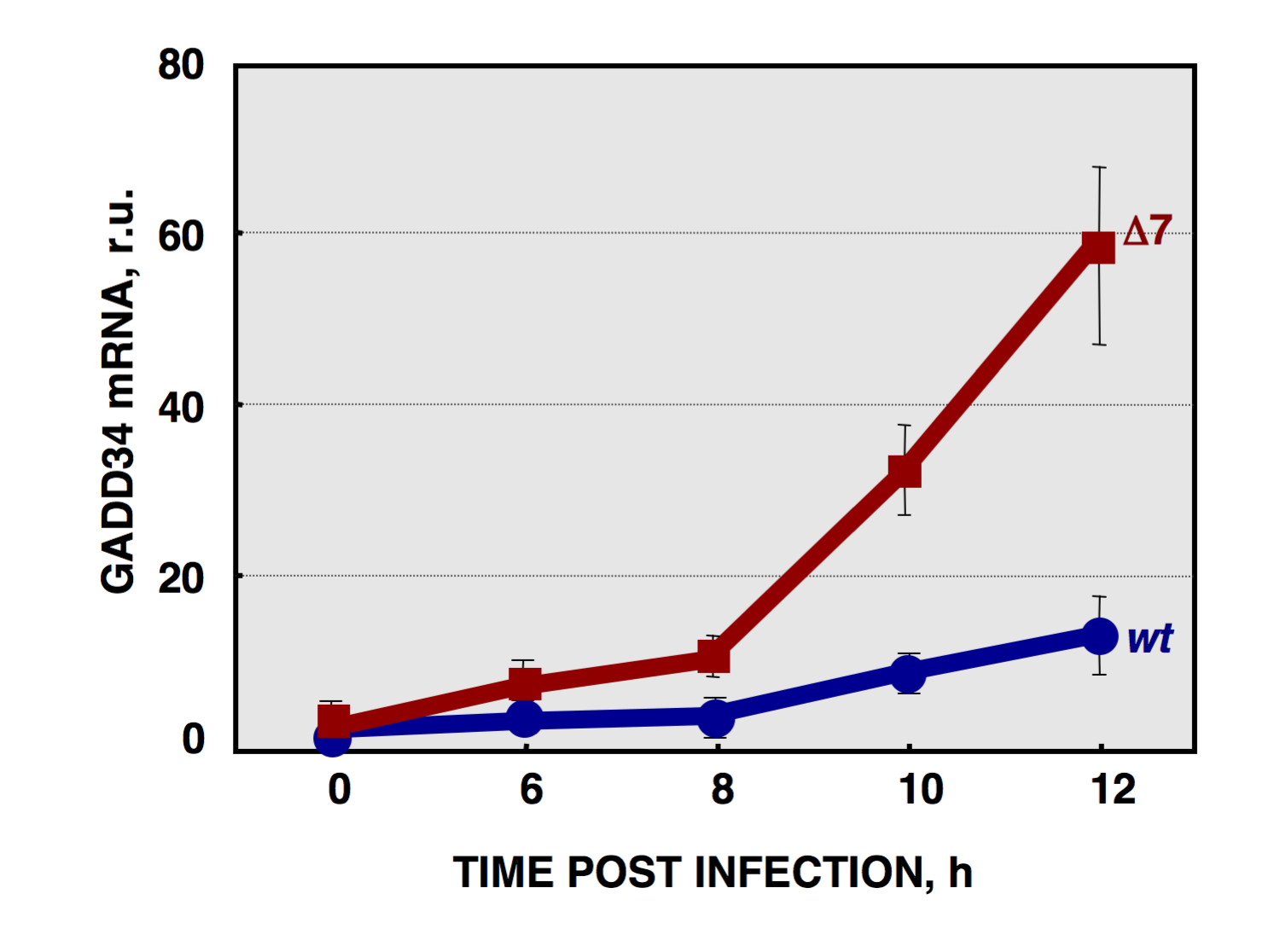

Supplement: Figure S1 — Porcine GADD34 expression. The expression of porcine GADD34, during rTGEV-wt (blue) or rTGEV-Δ7 (red) infections at indicated hpi, was analyzed by RT-qPCR. Error bars indicate the standard deviation from three independent experiments. r.u., relative units. (TIF) [file ppat.1002090.s001.tif]

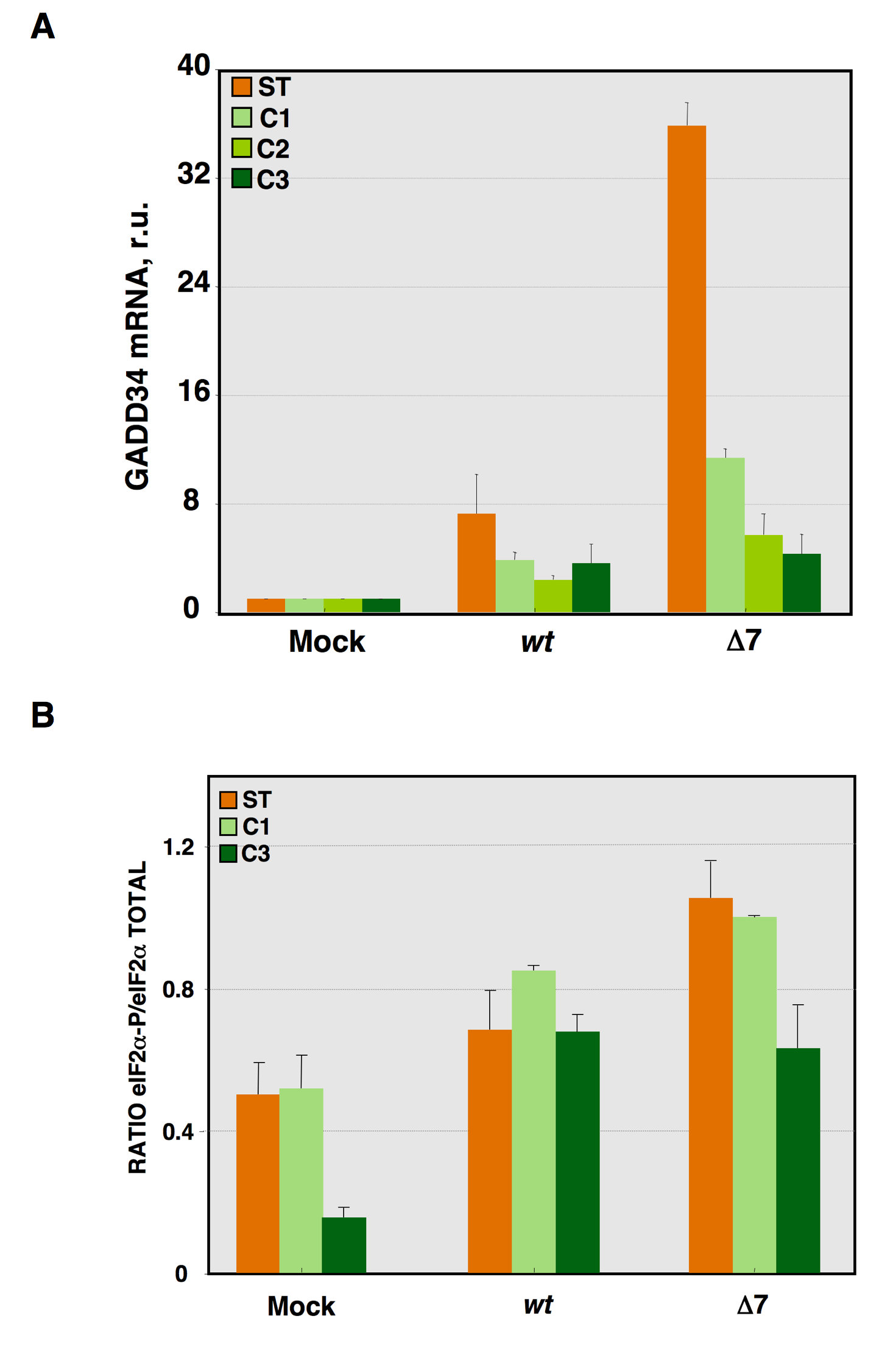

Supplement: Figure S2 — Decreased eIF2α-P by expression of TGEV protein 7 in trans. (A) ST cells and ST-HA-7 clones C1, C2 and C3 were infected with rTGEV-wt or rTGEV-Δ7. Total RNA was extracted at 10 hpi and porcine GADD34 expression was analyzed by RT-qPCR. r.u., relative units. Error bars represented the standard deviation of three independent experiments. (B) ST cells and ST-HA-7 clones C1 and C3 were infected with rTGEV-wt or rTGEV-Δ7. Total protein was extracted at 10 hpi and eIF2α and eIF2α-P protein levels were analyzed by Western-blot. Protein amounts were estimated by densitometry, and the ratio of eIF2α-P to total eIF2α was represented. Error bars represented the standard deviation of three independent experiments. (TIF) [file ppat.1002090.s002.tif]

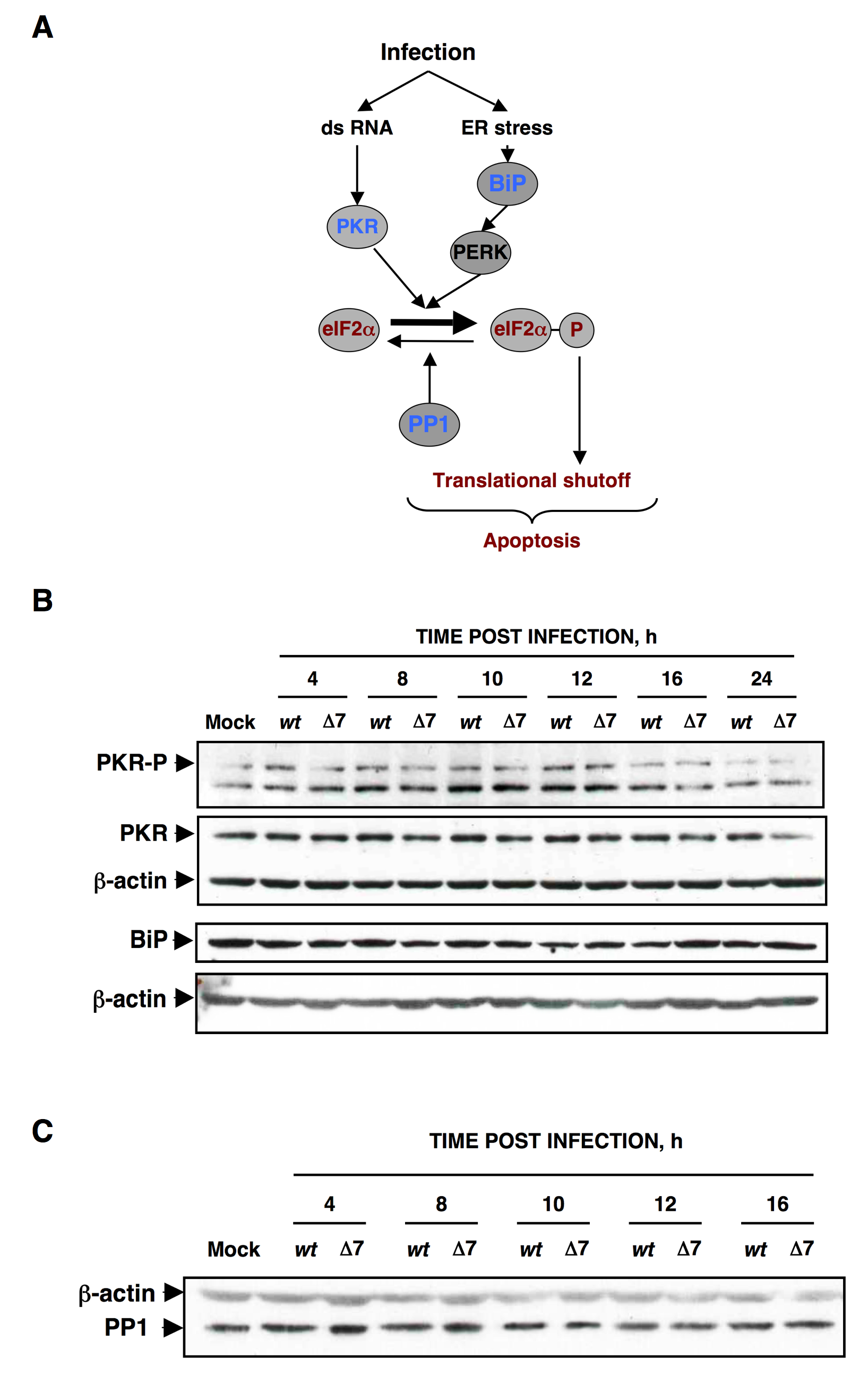

Supplement: Figure S3 — Effect of protein 7 on kinases implicated in eIF2α phosphorylation. (A) Scheme of eIF2α/eIF2α-P equilibrium influenced by PKR, PERK and PP1 activity. (B) Evaluation of phosphorylated PKR (PKR-P), total PKR and BiP accumulation during rTGEV-wt or rTGEV-Δ7 infections, at indicated hpi, by Western-blot using specific antibodies. β-actin was detected as loading control. (C) Analysis of PP1 accumulation in ST cells infected with rTGEV-wt or rTGEV-Δ7 at indicated times post infection, by Western-blot using a specific antibody. β-actin was detected as loading control. (TIF) [file ppat.1002090.s003.tif]

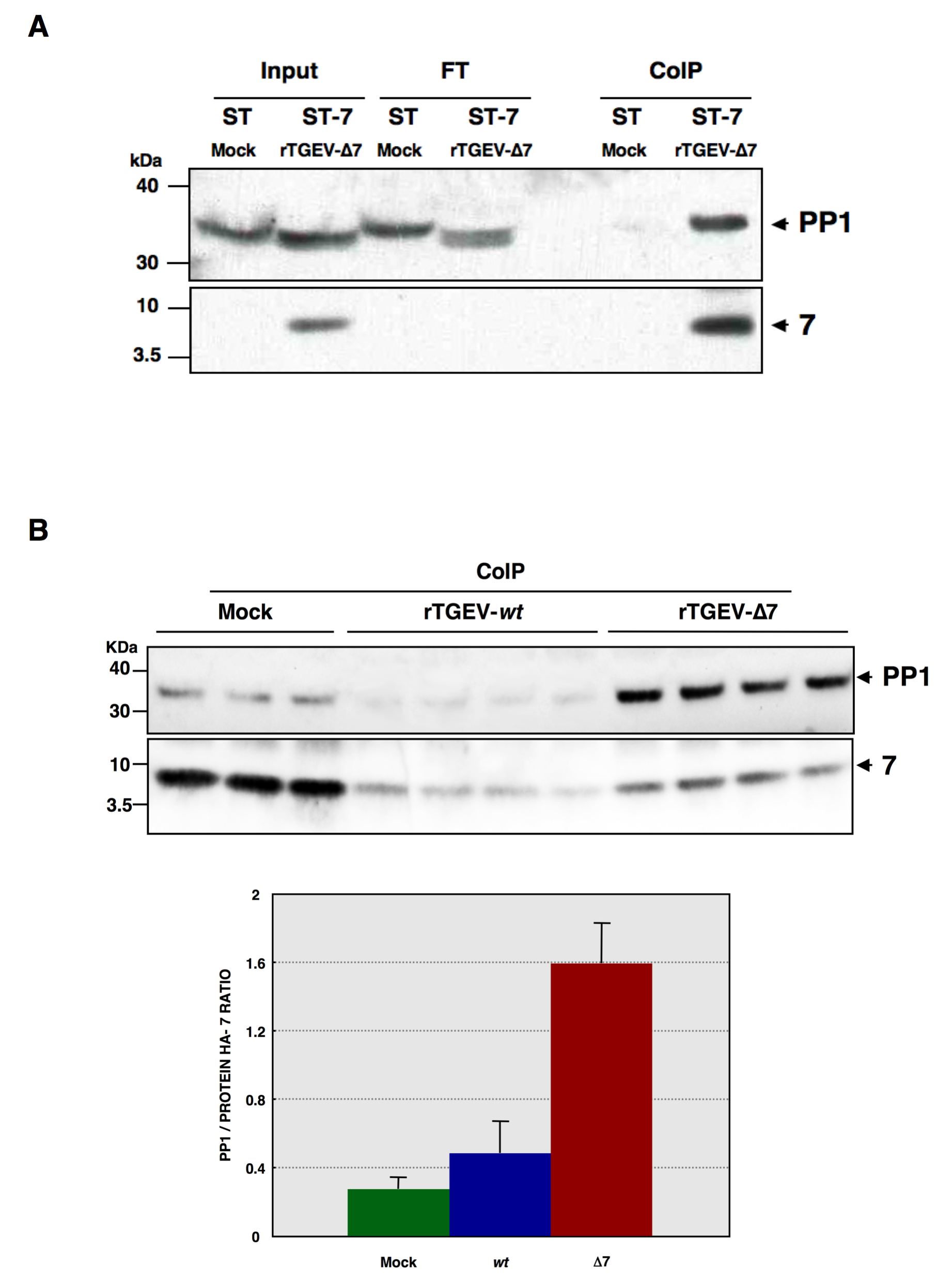

Supplement: Figure S4 — Interaction between PP1 and TGEV protein 7 in the context of TGEV infection. (A) ST mock infected cells, or ST-HA-7 cells infected with rTGEV-Δ7 were used for immunoprecipitation. Cell extracts from 16 hpi were incubated with anti-HA agarose. Input, flow through (FT), and final elution (CoIP) samples were resolved by SDS-PAGE. The presence of HA-tagged protein 7 and PP1 was analyzed by Western-blot using specific antibodies. (B) ST-HA-7 mock infected cells, or infected with rTGEV-wt or rTGEV-Δ7 viruses were used for immunoprecipitation as in (A). Co-immunoprecipitated (Co-IP) samples from different experiments were resolved by SDS-PAGE, and HA-tagged protein 7 and PP1 were detected by Western-blot. The graph represents the ratio between PP1 and HA-7 protein, estimated by densitometry. Error bars represent the standard deviation from the different experiments. (TIF) [file ppat.1002090.s004.tif]

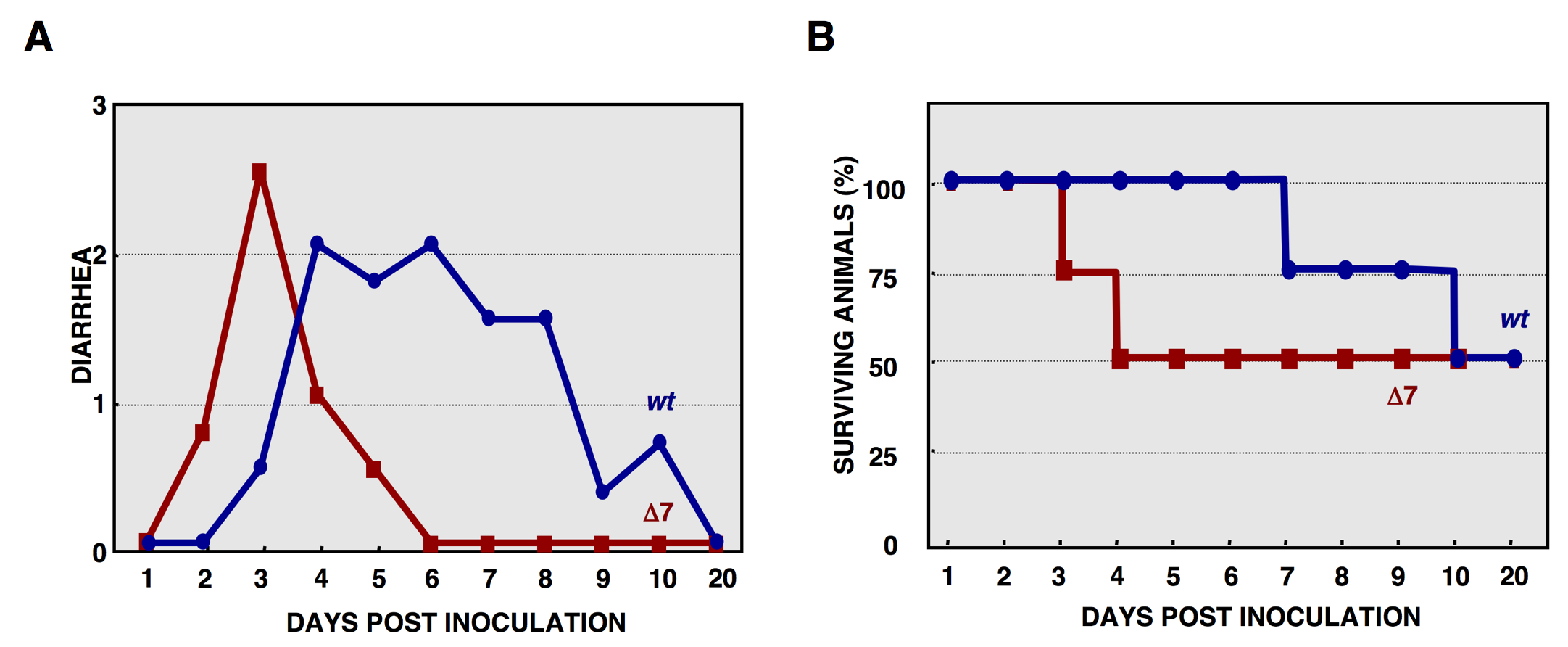

Supplement: Figure S5 — In vivo rTGEV-SC11-Δ7 virulence. Three-day-old piglets were inoculated with 1×10^7^ pfu/animal of rTGEV-SC11-wt or rTGEV-SC11-Δ7 viruses, by three routes (oral, intranasal and intragastric) in combination. (A) Clinical symptoms were analyzed during the experiment. The degree of diarrhea was represented: from 0, meaning healthy animal, to 3, meaning acute diarrhea. (B) Number of surviving piglets at different days post inoculation. (TIF) [file ppat.1002090.s005.tif]
